# **β** Cell–specific deletion of *Zfp148* improves nutrient-stimulated **β** cell Ca^2+^ responses

**DOI:** 10.1172/jci.insight.154198

**Published:** 2022-05-23

**Authors:** Christopher H. Emfinger, Eleonora de Klerk, Kathryn L. Schueler, Mary E. Rabaglia, Donnie S. Stapleton, Shane P. Simonett, Kelly A. Mitok, Ziyue Wang, Xinyue Liu, Joao A. Paulo, Qing Yu, Rebecca L. Cardone, Hannah R. Foster, Sophie L. Lewandowski, José C. Perales, Christina M. Kendziorski, Steven P. Gygi, Richard G. Kibbey, Mark P. Keller, Matthias Hebrok, Matthew J. Merrins, Alan D. Attie

**Affiliations:** 1 Department of Biochemistry, University of Wisconsin-Madison, Madison, Wisconsin, USA.; 2 UCSF Diabetes Center, UCSF, San Francisco, California, USA.; 3 Department of Biostatistics and Medical Informatics, University of Wisconsin-Madison, Madison, Wisconsin, USA.; 4 Biostatistics and Computational Biology Branch, National Institute of Environmental Health Sciences, Durham, North Carolina, USA.; 5 Department of Cell Biology, Harvard Medical School, Boston, Massachusetts, USA.; 6 Department of Internal Medicine (Endocrinology), Yale University, New Haven, Connecticut, USA.; 7 Department of Medicine, Division of Endocrinology, University of Wisconsin-Madison, Madison, Wisconsin, USA.; 8 Department of Physiological Sciences, School of Medicine, University of Barcelona, L’Hospitalet del Llobregat, Barcelona, Spain.; 9 Department of Cellular and Molecular Physiology, Yale University, New Haven, Connecticut, USA.; 10 William S. Middleton Memorial Veterans Hospital, Madison, Wisconsin, USA.

**Keywords:** Endocrinology, Metabolism, Beta cells, Calcium signaling, Insulin

## Abstract

Insulin secretion from pancreatic β cells is essential for glucose homeostasis. An insufficient response to the demand for insulin results in diabetes. We previously showed that β cell–specific deletion of *Zfp148* (β-*Zfp148*^KO^) improves glucose tolerance and insulin secretion in mice. Here, we performed Ca^2+^ imaging of islets from β‑*Zfp148*^KO^ and control mice fed both a chow and a Western-style diet. β-*Zfp148*^KO^ islets demonstrated improved sensitivity and sustained Ca^2+^ oscillations in response to elevated glucose levels. β-*Zfp148*^KO^ islets also exhibited elevated sensitivity to amino acid–induced Ca^2+^ influx under low glucose conditions, suggesting enhanced mitochondrial phosphoenolpyruvate-dependent (PEP-dependent), ATP-sensitive K^+^ channel closure, independent of glycolysis. RNA-Seq and proteomics of β-*Zfp148*^KO^ islets revealed altered levels of enzymes involved in amino acid metabolism (specifically, SLC3A2, SLC7A8, GLS, GLS2, PSPH, PHGDH, and PSAT1) and intermediary metabolism (namely, GOT1 and PCK2), consistent with altered PEP cycling. In agreement with this, β-*Zfp148*^KO^ islets displayed enhanced insulin secretion in response to l-glutamine and activation of glutamate dehydrogenase. Understanding pathways controlled by ZFP148 may provide promising strategies for improving β cell function that are robust to the metabolic challenge imposed by a Western diet.

## Graphical abstract

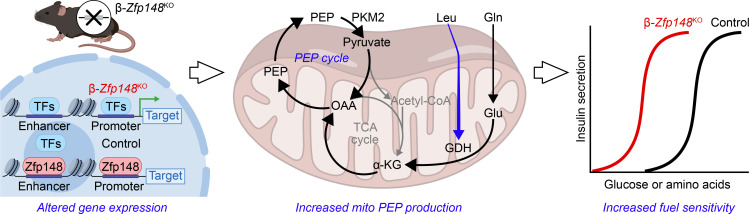

## Introduction

The genetic contribution to diabetes risk is primarily mediated through genetic variation in β cell function and β cell mass (1–3). Inbred mouse strains collectively contain as much genetic diversity as the human population, providing an ideal platform to screen for genes that affect diabetes-related traits. Diversity outbred (DO) mice are a stock of mice derived from interbreeding and outcrossing 8 inbred strains that include 5 commonly used mouse strains, and 3 wild-derived strains. With many generations of breeding, the DO mice have accumulated enough meiotic recombinations to map gene loci to within approximately 1 Mb resolution (4). To identify novel gene loci affecting insulin secretion, we performed a genetic screen of 500 DO mice (5). We identified multiple gene loci associated with circulating insulin levels following an oral glucose challenge and with insulin secretion measured ex vivo in isolated islets. A strong candidate gene for an insulin secretion quantitative trait locus on chromosome 16 is zinc finger protein 148 (*Zfp148*, also called *ZBP-89* and *BFCOL-1*, and, in humans, *ZNF148*) (5).

Whereas ZFP148 has previously defined roles in organ development (6–12), atherosclerosis (13), and cancer (14–20), a role in metabolism has been largely unexplored. We generated mice with a β cell–specific deletion of *Zfp148* (β-*Zfp148*^KO^; ref. 5). β-*Zfp148*^KO^ mice have dramatically improved glucose tolerance and insulin secretion, regardless of a metabolic challenge imposed by a Western-style diet (WD) (5). Here, we used Ca^2+^ imaging to identify the mechanisms linking deletion of β cell *Zfp148* to enhanced insulin secretion. We show that islets from β-*Zfp148*^KO^ mice have markedly enhanced sensitivity to glucose, as revealed by Ca^2+^ dynamics, compared with islets of littermate controls. Nonglycolytic stimulators of Ca^2+^ signaling and insulin secretion, coupled with transcriptomics and proteomics, suggest this heightened sensitivity to glucose is mediated by increased phosphoenolpyruvate (PEP) cycling, a known regulator of insulin secretion (21–23) and a promising new therapeutic target (22, 23).

## Results

### β-Zfp148^KO^ islets demonstrate increased responsivity to glucose.

Insulin secretion is pulsatile (24–27). β Cells demonstrate oscillations in both cytosolic Ca^2+^ and metabolism that are intrinsically linked to the amplitude and kinetics of insulin secretion (25, 28–32). Alterations in slow, oscillatory Ca^2+^ responses of islets to glucose can reveal metabolic mechanisms regulating insulin secretion (22, 33–35).

**Figure 1:**
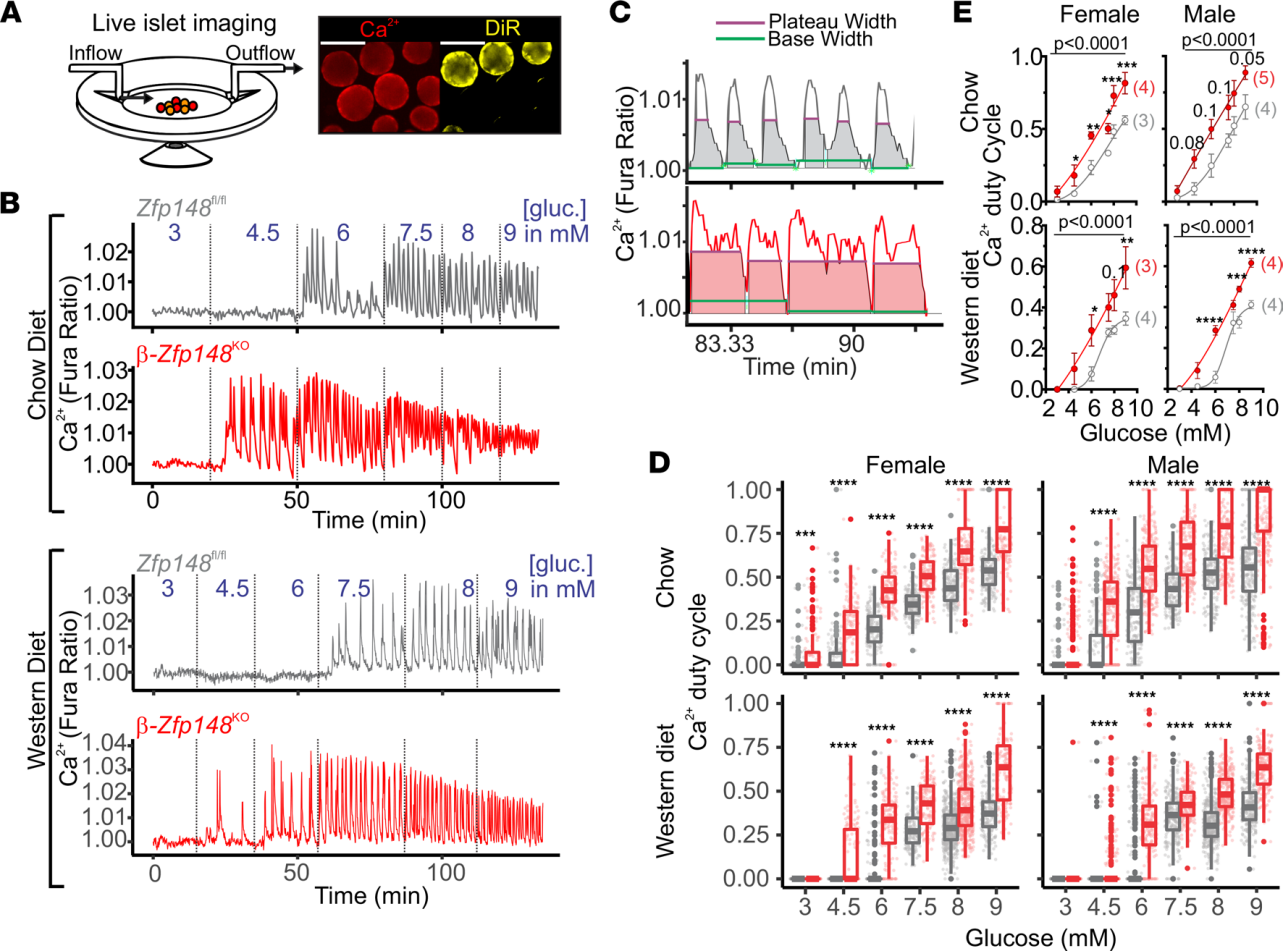
β-Zfp148^KO^ islets show improved glucose sensitivity and altered Ca^2+^ duty cycle. (**A**) Islets from 2 animals were imaged simultaneously during perifusion (left) by labeling 1 animal’s islets with the lipid dye DiR (right; scale bar: 100 μm). (**B**) Detrended example traces from simultaneously imaged *Zfp148^fl/fl^* control and β*-Zfp148*^KO^ islets using Fura Red. Islets from chow-fed (top) and WD-fed mice (bottom) were imaged during a glucose (gluc) ramp (3, 4.5, 6, 7.5, 8, and 9 mM glucose; transition times shown by dotted lines). (**C**) MATLAB scripts measure base and peak width for oscillations to determine duty cycle in *Zfp148^fl/fl^* control and β*-Zfp148*^KO^ islets. (**D**) By-islet duty-cycle measurements from islets of female (left) and male (right) *Zfp148^fl/fl^* control and β*-Zfp148*^KO^ mice fed chow (top) and WD (bottom). Dots indicate individual islet trace values for each condition and gene. Overlaid box plots show summary data (top and bottom edges indicate 25th and 75th percentile, respectively; the 50th percentile is indicated by the midline, and lines on either side mark 1.5× the IQR). Statistical significances (Šidák post tests after 2-way ANOVA, corrected for multiple comparisons) are indicated. (**E**) By-animal dose-response curves derived from average duty-cycle measurements for β-*Zfp148*^KO^ and littermate control mice for the conditions in **D**. The number of animals analyzed per group is indicated in parentheses. Mean ± SEM are plotted, with the line representing the sigmoidal dose-response curve determined using PRISM. Statistical comparisons of fitted curves are shown above the curves, as determined using extra sum-of-squares *F* test. (**D** and **E**) *adjusted *P* < 0.05, **adjusted *P* < 0.01, ***adjusted *P* < 0.001, and ****adjusted *P* < 0.0001. Subthreshold *P* values for point comparisons in **E** are indicated above the relevant points. These data were derived from ≥140 islets from ≥3 mice per sex per diet per genotype. Islet and animal distributions per group are in listed in Table 1.

We analyzed real-time Ca^2+^ oscillations of islets from β-*Zfp148*^KO^ and littermate control mice in response to increasing glucose concentrations (Figure 1). Our experimental design (Figure 1A) allowed us to simultaneously measure Ca^2+^ responses in islets from β-*Zfp148*^KO^ and control mice using 1,1′-dioctadecyl-3,3,3′,3′-tetramethylindotricarbocyanine iodide (DiR) labeling of islets from 1 mouse (see Methods). Glucose-evoked Ca^2+^ oscillations in β-*Zfp148*^KO^ islets were initiated at a lower glucose concentration (4.5 mM) than in control islets (6 mM), reflecting the increased glucose sensitivity of the β-*Zfp148*^KO^ islets (Figure 1, B–D). Islet Ca^2+^ duty cycle (the fraction of time during which the Ca^2+^ level is elevated while oscillating; Figure 1C) was increased in the β-*Zfp148*^KO^ islets at all glucose concentrations (Figure 1, D and E). Islets collected from mice maintained on a WD high in fat and sucrose showed reduced oscillatory duty-cycle Ca^2+^. However, the leftward shift in the Ca^2+^ duty-cycle response curve for β-*Zfp148*^KO^ islets was observed in both chow- and WD-fed mice (Figure 1E and Supplemental Figure 1; supplemental material available online with this article; https://doi.org/10.1172/jci.insight.154198DS1).

### β-Zfp148^KO^ islets show enhanced mitochondrial PEP-dependent Ca^2+^ influx.

Glutamate dehydrogenase (GDH) plays a key role in amino acid–induced stimulation of insulin secretion (36). β Cells express high levels of mitochondrial phosphoenolpyruvate carboxykinase (*Pck2*), and deletion of this gene dramatically reduces insulin secretion and blocks amino acid–stimulated Ca^2+^ influx at low glucose levels (23, 37). Activators of pyruvate kinases stimulate insulin secretion (22). Together, these observations point to a pathway whereby formation of mitochondrial PEP and its pyruvate kinase-dependent cycling back to pyruvate play a key role in insulin secretion. By measuring Ca^2+^ responses to amino acids at a nonstimulatory concentration of glucose (e.g., 3 mM), we asked if mitochondrial PEP contributes to improved insulin secretion of β-*Zfp148*^KO^ islets. Under these conditions, amino acids feed into the TCA cycle via GDH, providing oxaloacetate (OAA) for the PCK2 reaction independently of input from glycolytic intermediates (see below and also ref. 22). Thus, with a restricted glycolytic PEP supply, the PEP substrate used by pyruvate kinase to generate the ATP that closes the ATP-sensitive K^+^ (K_ATP_) channel derives predominantly, if not exclusively, from mitochondrial PEP production via PCK2 (23, 37).

**Figure 2:**
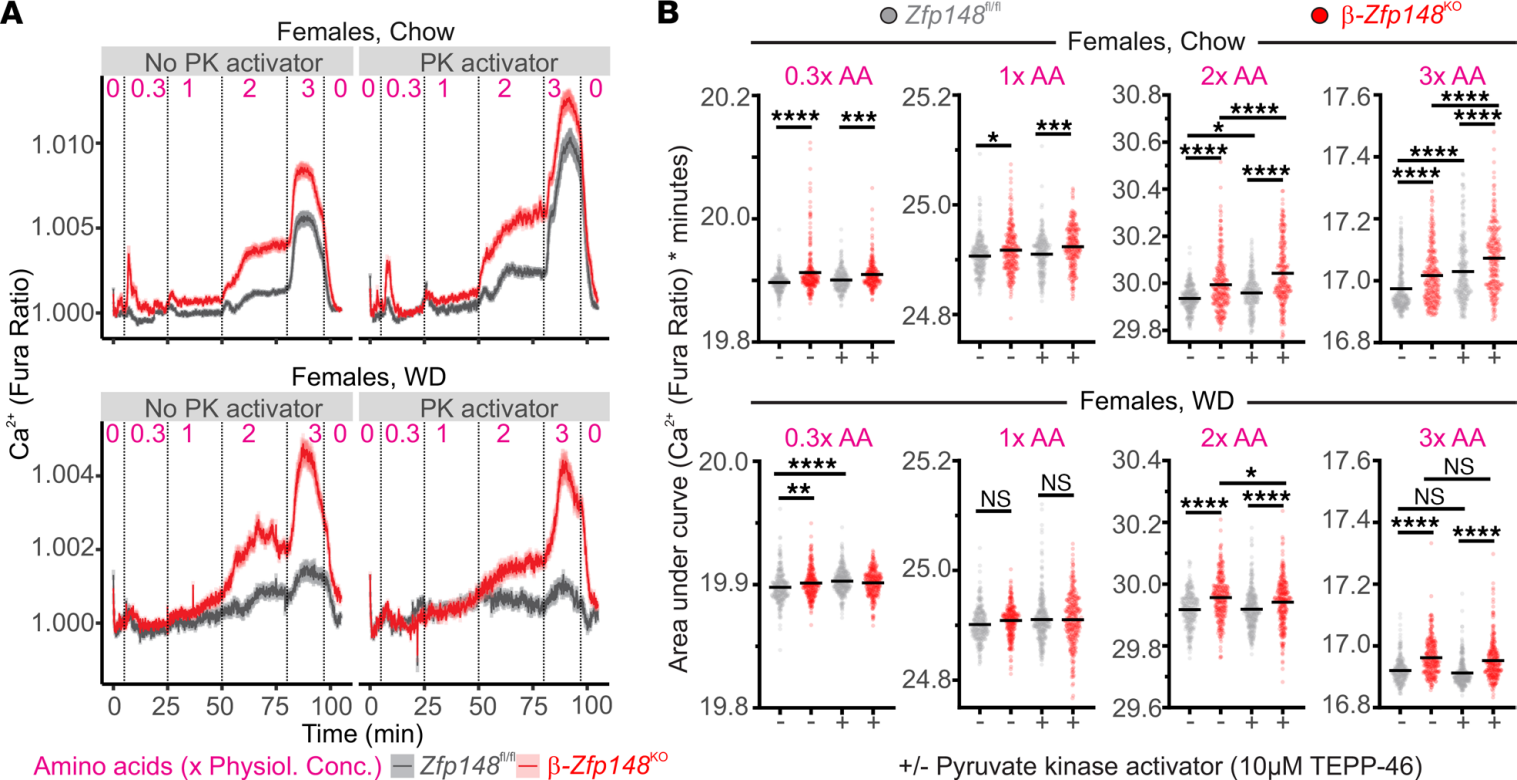
β-Zfp148^KO^ islets show altered amino acid sensitivity. (**A**) Average Ca^2+^ traces from islets of female chow-fed (top) and WD-fed (bottom) β-*Zfp148*^KO^ and littermate control mice during amino acid (AA) ramps (numbers above the traces indicate concentration of the 12 amino acids in the mix relative to their physiological concentrations (physiol. conc.; e.g., 1× indicates physiological levels in plasma) with and without pyruvate kinase (PK) activator (10 μM TEPP-46). All solutions contained 3 mM glucose. Dotted lines indicate transitions between solutions. (**B**) AUC analysis of islet traces during the amino acid ramps. Concentrations of the amino acids are indicated above the graphs. Data in the top and bottom panels are from the chow-fed and WD-fed mouse islets, respectively. There are ≥237 islets from 5–7 mice per group per diet. The islet and animal distributions for each group are indicated in Table 1. (**B**) Data from individual islet traces are indicated as points and the lines indicate the mean; *P < 0.05, **P < 0.01, ***P < 0.001, and ****P < 0.0001. Data were analyzed by Tukey post tests after 1-way ANOVA and *P* values were adjusted for multiple comparisons.

The Ca^2+^ responses in the female β-*Zfp148*^KO^ islets were much more sensitive to amino acids, even at concentrations that did not increase Ca^2+^ in control islets (Figure 2, A and B, and Supplemental Figure 2), analogous to elevated glucose sensitivity. These results are consistent with increased mitochondrial PEP production, and/or increased pyruvate kinase activity in β-*Zfp148*^KO^ islets. The enhanced sensitivity to amino acids was also observed in mice fed the chow diet and those fed the WD, although the WD dampened the response to amino acids in both WT and β-*Zfp148*^KO^ islets (Figure 2, A and B, and Supplemental Figure 2).

Ca^2+^ responses evoked by amino acids plus low glucose levels probe PEP cycling mediated by mitochondrial-derived PEP. However, the enhanced Ca^2+^ response observed for β-*Zfp148*^KO^ islets in response to these conditions could reflect altered pyruvate kinase activity or OAA production via anaplerotic flux. TEPP-46 is a small molecule activator of the M2 and L isoforms of pyruvate kinase (PKM2 and PKL, respectively; refs. 22 and 38). To distinguish the contribution of anaplerotic flux from activation of pyruvate kinase, we treated β-*Zfp148*^KO^ and control islets with TEPP-46 during exposure to low-glucose plus amino acids. If the loss of *Zfp148* led to increased Ca^2+^ through enhanced production of OAA, rather than increased pyruvate kinase activity, then β-*Zfp148*^KO^ islets would be expected to retain an elevated Ca^2+^ response in the presence of TEPP-46. Activating pyruvate kinase with TEPP-46 increased Ca^2+^ responses to amino acids in both β-*Zfp148*^KO^ and control islets, as has been shown for other mice (22, 23). However, β-*Zfp148*^KO^ islets had elevated Ca^2+^ levels compared with control islets (Figure 2, A and B, and Supplemental Figure 2), consistent with increased mitochondrial anaplerotic OAA production. This was also observed in islets from WD-fed mice, although the pyruvate kinase activator did not have as strong an effect to increase amino acid–stimulated Ca^2+^ responses in the female mice (Figure 2, A and B, and Supplemental Figure 2).

Consistent with the individual islet average traces shown in Figure 2 and Supplemental Figure 2, the average Ca^2+^ responses evoked by amino acids for all islets from a mouse were increased in β-*Zfp148*^KO^ mice (Supplemental Figure 2). Islets from WD-fed male β-*Zfp148*^KO^ mice had remarkably increased Ca^2+^ responses to lower concentrations of amino acids (Supplemental Figure 3), an effect preserved even with pyruvate kinase activation. Islets from chow-fed male mice showed trends consistent with increased Ca^2+^ responses to lower concentrations of amino acids in β-*Zfp148*^KO^ islets (Supplemental Figures 2 and 3). Collectively, these data demonstrate enhanced amino acid–induced Ca^2+^ responses in the β-*Zfp148*^KO^ islets, regardless of sex or dietary challenge.

### Loss of Zfp148 alters islet expression of multiple metabolic genes.

**Figure 3:**
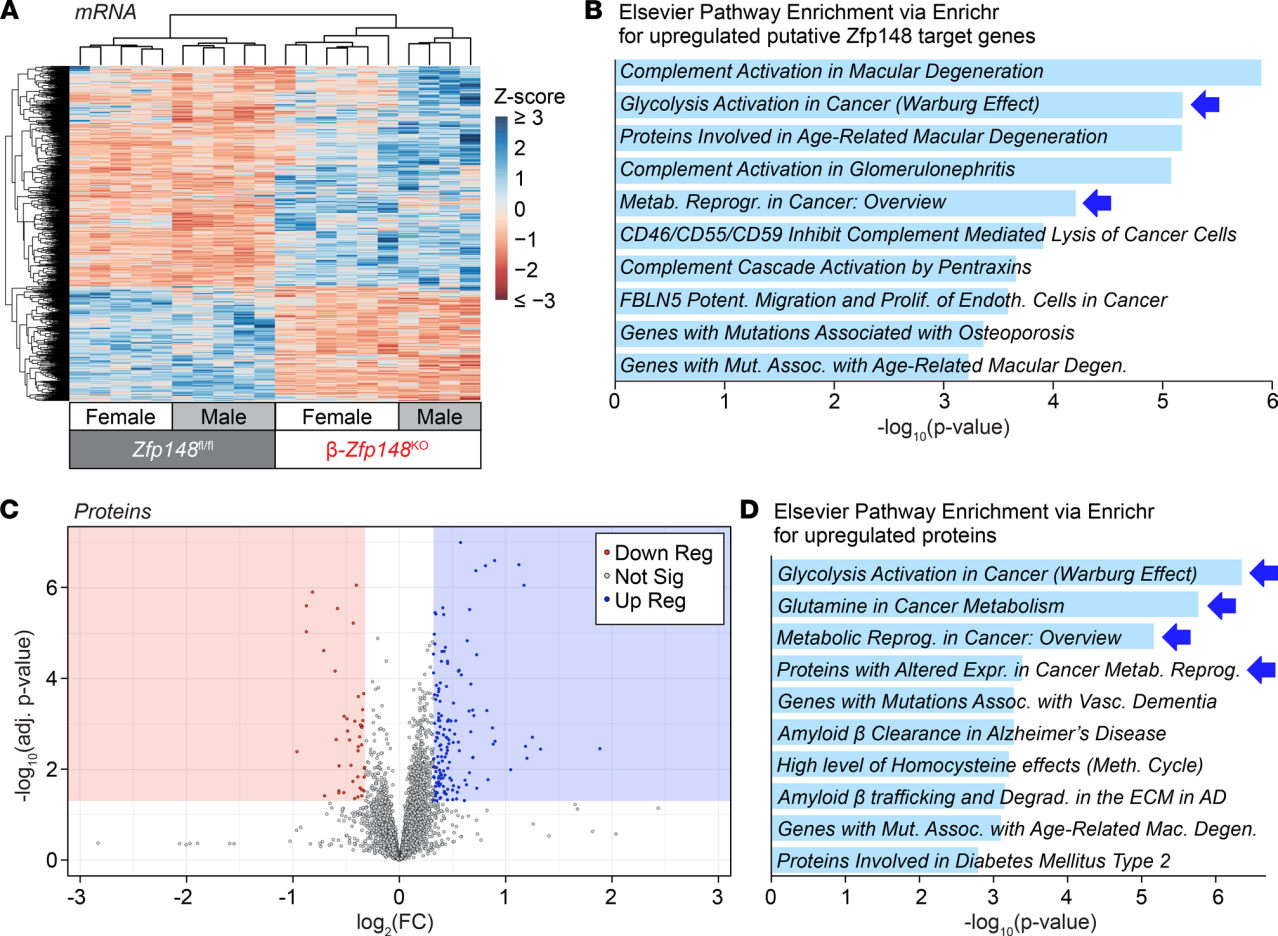
Altered expression of metabolic genes and proteins in β-Zfp148^KO^ and control islets. (**A**) Heat map of *z*-scores for significantly DE genes in RNA-Seq from islets of 10 WD-fed *Zfp148^fl/fl^* control and β-*Zfp148*^KO^ mice, as determined using EBSeq. (**B**) Elsevier database pathway enrichment (from Enrichr; refs. 46 and 47) for upregulated, putative, direct DE targets of Zfp148, based on analysis in Supplemental Figure 4. (**C**) Volcano plot of proteins significantly increased or decreased in islets of 6 WD-fed β-*Zfp148*^KO^ vs. 10 WD-fed *Zfp148^fl/fl^* control mice. (**D**) Elsevier database pathway enrichment for proteins significantly increased in β-*Zfp148*^KO^ islets. For pathway enrichments, terms in italics indicate enrichment beyond the false discovery cutoff (adjusted *P* < 0.05). Corresponding genes for the indicated terms are listed in extended data in Dryad (46). (**B** and **D**) Blue arrows indicate related enrichment terms from both RNA and protein data sets. AD, Alzheimer disease; adj, adjusted; assoc, associated; degen, degeneration; degrad, degradation; ECM, extracellular matrix; endoth, endothelial; expr, expression; FC, fold change; mac, macular; metab, metabolic; meth, methionine; mut, mutations; potent, potentiates; prolif, proliferation; reprog, reprogramming; sig, significant; vasc, vascular.

To further explore the links between ZFP148’s role as a transcription factor and enhanced glucose and amino acid–induced Ca^2+^ responses in β-*Zfp148*^KO^ islets, we performed RNA-Seq on islets from male and female β-*Zfp148*^KO^ and littermate control mice fed a WD (Figure 3A). We identified more than 600 transcripts that were differentially expressed (DE) between β-*Zfp148*^KO^ and control islets, with approximately 400 that were increased in β-*Zfp148*^KO^ islets, consistent with a known role of ZFP148 as a transcriptional repressor in other tissues (39–43).

ZFP148 may alter chromatin architecture at specific binding sites. ZFP148 may bind near transcription start sites (TSSs) for its target genes, within their introns, or may alter links between the gene’s TSS and known islet enhancer regions. To interrogate this, we aligned the orthologs of DE genes identified in Figure 3A across existing ZNF148 ChIP-Seq data from other cell types (44) and islet chromatin data (assay for transposase-accessible chromatin–Seq, methylation, acetylation, HiC, and annotated enhancers; ref. 45). In addition to those genes with ZNF148 binding sites within 1 kbp of their TSS or within their introns (e.g., *PDK4, PSPH,* and *VOPP1*; Supplemental Figure 4, A and B), some had ZNF148 binding sites within 500 bp of HiC loops connecting to known enhancer regions (Supplemental Figure 4, A and B). For example, the ZNF148 binding sites near *PSPH* (Supplemental Figure 4B) align with looping between the *PSPH* promoter and a known islet “super-enhancer” region (45). Overall, these alignments revealed more than 200 DE gene orthologs that may be targets of ZNF148, either through binding of ZNF148 near the genes (TSS or introns) or chromatin looping. As might be expected, DE genes that are predicted ZFP148 targets contained motifs for ZFP148 and for its known binding partners or competitors (e.g., ZFP281, SP1, and SP3; extended data in Dryad, ref. 46) (47, 48). Additionally, the promoters for a handful of these candidates, including *Psph* and *Pdk4*, bind to ZFP148 in mouse cell lines (49). The predicted direct transcriptional targets of ZFP148 suggest a possible mechanism to explain the altered glucose and amino acid sensitivity of the β-*Zfp148*^KO^ islets.

Islet Ca^2+^ dynamics are intrinsically linked to the activity of K_ATP_ channels (24). Neither of the genes encoding the K_ATP_-channel subunits *(Abcc8* and *Kcnj11*) was differentially expressed in β-*Zfp148*^KO^ versus control islets, suggesting that the altered Ca^2+^ signals may be due to changes upstream of K_ATP_ channels. Gene ontology enrichment for either the complete DE gene set or for those genes with the greatest fold change (>2.5-fold) revealed associations with extracellular matrix proteins and proteins with transaminase activity, among others (Supplemental Figures 5, A and B, and extended data in Dryad, ref. 46). Additionally, pathway analysis for potential direct targets revealed enrichments for metabolic adaptations in cancers (Figure 3B), diseases for which ZFP148 has known roles (16, 17), and included *Psat1* and *Psph*, which may result in altered activity of enzymes (e.g., PKM2; Figure 3B and extended data in Dryad, ref. 46), which have roles in both cancer and insulin secretion (22, 50). Other DE genes possibly involved in metabolic adaptations in β-*Zfp148*^KO^ islets included genes involved in amino acid transport and metabolism. Pathway analysis for the decreased putative ZFP148 targets did not show any terms meeting the significance cutoff threshold (Supplemental Figure 4D and extended data in Dryad, ref. 46).

**Figure 4:**
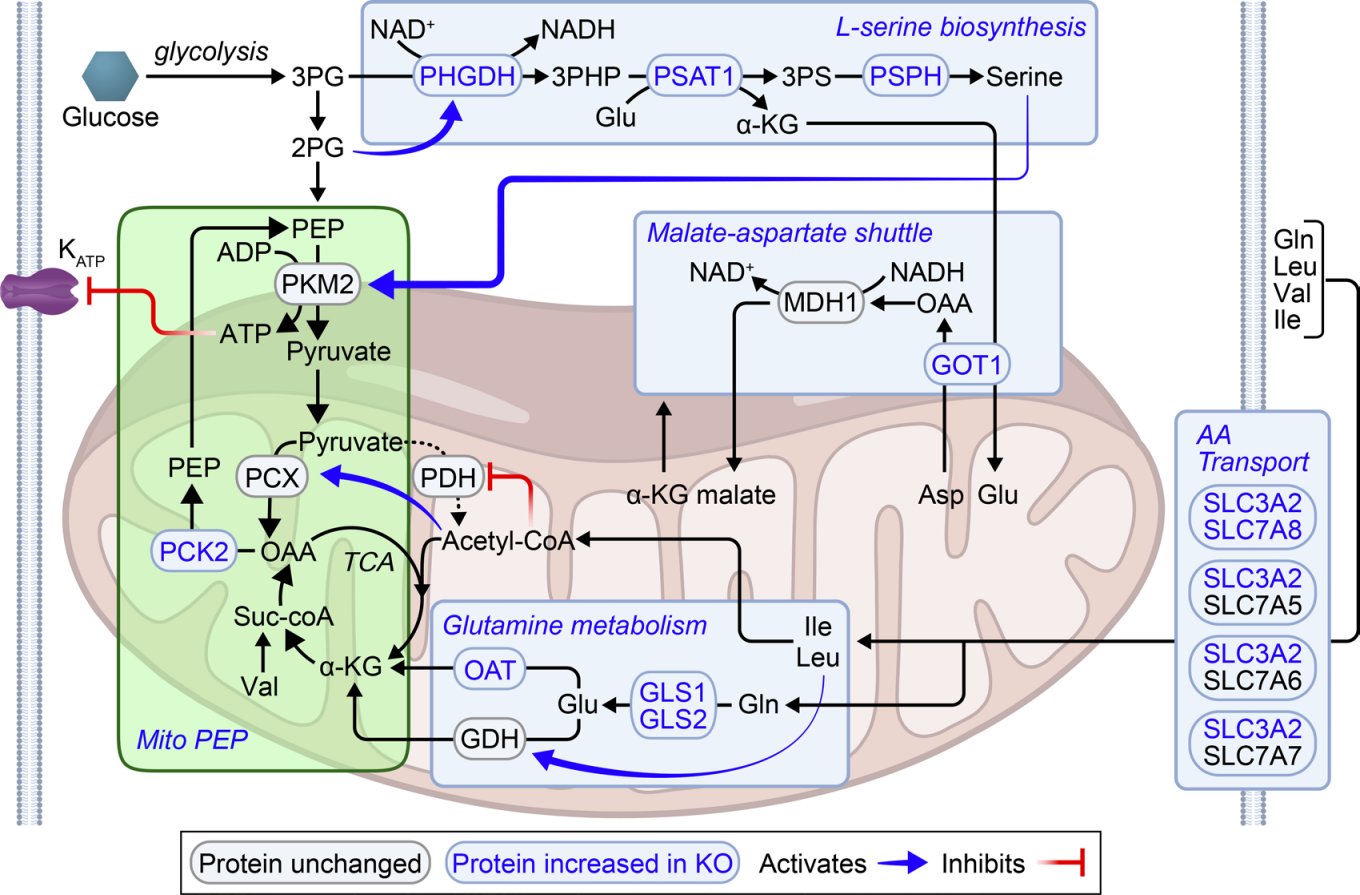
Possible pathway for enhanced mitochondrial PEP production and cycling in β-Zfp148^KO^ islets. Pyruvate generated from PEP can either be converted to acetyl-coA by pyruvate dehydrogenase (PDH) and enter the TCA cycle or it can be converted to OAA by pyruvate carboxylase (PCX). OAA can be converted into PEP via PCK2. PEP can then be converted back to pyruvate by pyruvate kinase (PKM2), completing a PEP cycle. Conversion of PEP into pyruvate by PKM2 generates the ATP, which closes the K_ATP_ channels and triggers membrane depolarization, Ca^2+^ entry, and insulin release. Proteins increased in β-*Zfp148*^KO^ islets vs. control islets include enzymes involved in amino acid metabolism and mitochondrial PEP production. In the proposed model, altered amino acid (AA) metabolism intersects mitochondrial PEP production and cycling at key points (highlighted in light blue boxes). Initially, increased production of l-serine results in increased activation of PKM2. Increased GOT1 may mediate regeneration of NAD^+^ for glycolysis and serine biosynthesis via the malate–aspartate shuttle. Additionally, key genes involved in AA metabolism result in increased substrate flux to the TCA cycle via α-ketoglutarate and acetyl-coA. Increased AA transporters (SLC3A2/SLC7A8, and others) mediate influx of Gln, Leu, Val, and Ile. Ile and Leu produce acetyl-coA, resulting in increased inhibition of PDH and activation of PCX. Valine metabolism may result in increased succinyl-coA. Finally, increased levels of several enzymes may mediate increased production of α-ketoglutarate from Glu and Gln, a process enhanced by Leu activation of GDH. The α-ketoglutarate then provides substrate for producing OAA, which is then converted to PEP by increased PCK2. Collectively, metabolic genes altered by loss of *Zfp148* in β cells may thereby contribute to enhanced mitochondrial PEP production and cycling, resulting in enhanced insulin secretion.

To confirm whether pathways predicted to be altered in the β-*Zfp148*^KO^ islets’ RNA-Seq translated to differential protein expression for the key enzymes linked to these metabolic pathways, we performed unbiased proteomics on islets from 10 WD-fed control and 6 WD-fed β-*Zfp148*^KO^ male mice. Of the more than 6000 proteins identified, 148 were significantly increased (fold change > 1.25 and corrected *P* < 0.05) and 44 were significantly decreased (Figure 3C). Although proteins decreased in the β-*Zfp148*^KO^ islets enriched for mRNA and transcriptional processing proteins, no Elsevier pathway analysis enrichment terms met the significance cutoff threshold (Supplemental Figure 4E and extended data in Dryad, ref. 46). Additionally, consistent with the RNA-Seq analysis, neither K_ATP_ channel subunit showed differential protein abundance. Strikingly, the top Elsevier pathway analysis enrichment terms for significantly increased proteins included metabolic adaptation in cancer, activation of glycolysis in cancer, amino acid metabolism, and type 2 diabetes, mirroring some of the terms for the RNA-Seq analysis (Figure 3D, similar terms in Figure 3, B and D are highlighted with blue arrows). The pathways in which these proteins function may intersect (Figure 4) to enhance mitochondrial PEP production and cycling, a key regulator of K_ATP_ channel closure and insulin secretion (22).

### β-Zfp148^KO^ islets show enhanced mitochondrial PEP-dependent insulin secretion.

**Figure 5:**
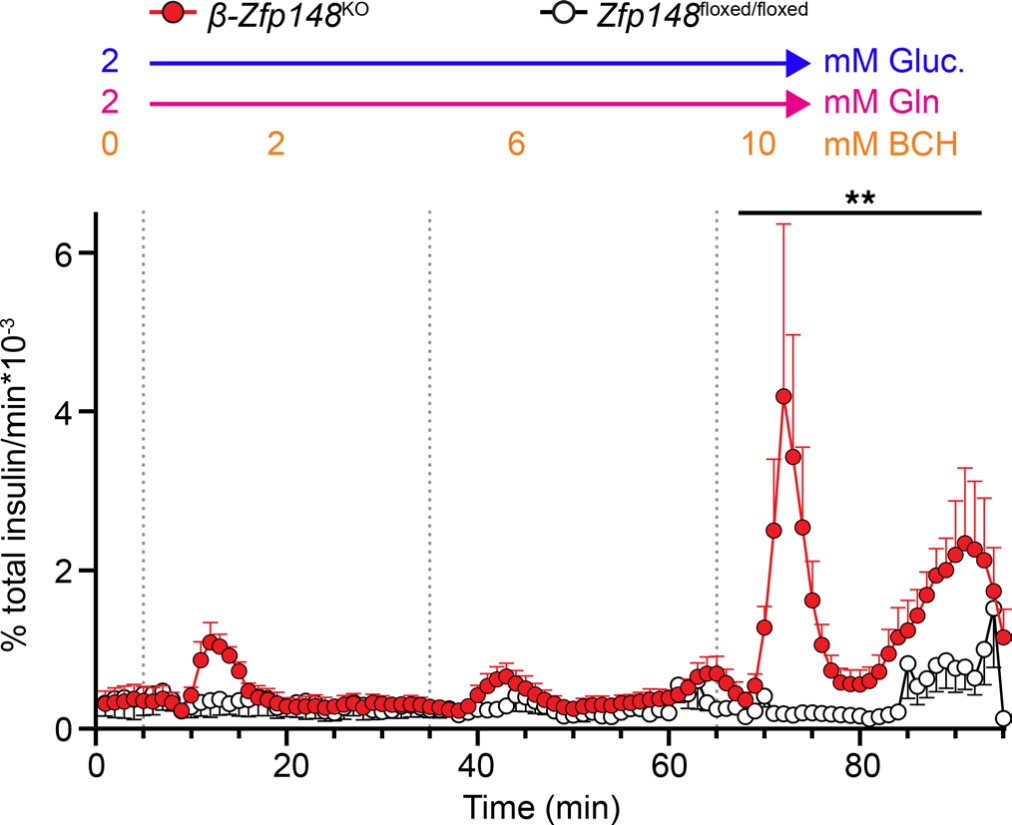
Enhanced insulin secretion from mitochondrial PEP in β-Zfp148^KO^ islets. Fractional secretion from islets of chow-fed male β-*Zfp148*^KO^ (red circles and traces) and control (open circles, black traces) mice during a low-glucose ramp of GDH activator BCH in perifusion. Dotted vertical lines indicate transitions between the different solutions. All solutions contained 2 mM glucose (Gluc) and 2 mM l-glutamine (Gln). Concentration of BCH is indicated above each respective segment of the perifusion traces. *n* = 4/genotype. **P < 0.01, Šidák post test after 2-way ANOVA of AUC of the segment, β-*Zfp148*^KO^ vs. control islets. Trace points display mean ± SEM. There was no difference in insulin content per islet between groups (data not shown).

One prediction of our model in Figure 4 is that β-*Zfp148*^KO^ islets would have greater production of PEP from intermediates entering the TCA cycle at α-ketoglutarate and producing increased OAA. However, some of the amino acids used in the Ca^2+^ imaging experiments (Figure 2) can also act through nonmetabolic pathways (51, 52). Glutamine is converted to glutamate, which can be converted to α-ketoglutarate and enter the TCA cycle to produce OAA, and indeed, 1 term in the protein pathway analysis was glutamine metabolism. However, glutamine is normally insufficient by itself to stimulate insulin secretion with low glucose (53–55). GDH, the enzyme that converts glutamate to α-ketoglutarate, is normally inhibited by GTP produced during TCA cycling, an effect overcome using the nonmetabolized leucine analog 2-amino-2-norbornanecarboxylic acid (BCH) (53, 54). To determine whether production of PEP from intermediates entering at α-ketoglutarate influenced insulin secretion independently of other signals, we exposed islets of chow-fed, male β-*Zfp148*^KO^ and control mice to a perifusion of 2 mM glucose, 2 mM l-glutamine, and increasing concentrations of BCH (Figure 5 and Supplemental Figure 6). Consistent with our model, β-*Zfp148*^KO^ clearly demonstrated enhanced insulin secretion in response to 10 mM BCH and l-glutamine, whereas very little was secreted by controls in this condition.

## Discussion

We previously showed that mice with a β cell–specific loss of *Zfp148* display enhanced glucose tolerance and elevated insulin secretion from cultured islets in response to a variety of secretagogues (5). Here, we show that β-*Zfp148*^KO^ islets have a lower threshold for glucose-induced Ca^2+^ elevation and a prolonged Ca^2+^ oscillatory duty cycle. RNA-Seq of islets from β-*Zfp148*^KO^ and control mice identified numerous genes that control nutrient responses. We demonstrated that 1 or more of these pathways converge to enhance PEP cycling and, consequently, pyruvate kinase–derived ATP production (Figures 2 and 5, and Supplemental Figures 2 and 3), the direct activator of K_ATP_ channel closure and a known regulator of insulin secretion (22, 37).

### ZFP148 alters islet metabolic cycling.

ZFP148 has been studied for its roles in various cancers (14–19, 49) and organ development (7–9, 11, 12). A role for ZFP148 in metabolism has received much less attention. ZFP148 regulates cytochrome c oxidase Vb and cyclin D1 during myocyte differentiation (10, 43). ZFP148 competes with SP1 and thereby regulates expression of IRS2 in some neurons (56). It also regulates fatty acid–induced gene expression in INS-1 cells (57) and is highly expressed in multiple islet endocrine cell types (58).

In addition to glucose, amino acids stimulate insulin secretion. The role of GDH in this pathway was revealed through the discovery that people with mutations resulting in constitutively active GDH have hyperinsulinemia and hyperammonemia (36, 59). Together with subsequent work demonstrating a critical role for mitochondrial PEPCK (encoded by *Pck2*) in amino acid–stimulated insulin secretion, the formation of PEP and its cycling via PK compose the pathway mediating amino acid–stimulated insulin secretion (22, 23, 37). The enhanced sensitivity to amino acid–induced Ca^2+^ elevation we observed in β-*Zfp148*^KO^ islets is consistent with elevated PEP cycling.

Several enzymes playing key roles in intermediary metabolism were increased in islets from β-*Zfp148*^KO^ mice. Pyruvate derived from PEP can either be converted to acetyl-CoA via pyruvate dehydrogenase and enter the TCA cycle or to OAA via pyruvate carboxylase (PCX) (Figure 4). OAA is converted to PEP by PCK2, which is highly abundant in β cell mitochondria. PEP is exported to the cytosol where it is converted to pyruvate (completing a PEP cycle) by the 3 isoforms of pyruvate kinase (PKM1, PKM2, and PKL), generating ATP, which closes K_ATP_ channels, triggering Ca^2+^ influx and insulin release (22, 23). Both our RNA-Seq and proteomic analyses of β-*Zfp148*^KO^ and control islets point to pathways that may enhance this process in the cytosol and in the mitochondria (Figure 4).

In the cytosol, according to our model, increased phosphoglycerate dehydrogenase, phosphoserine phosphatase (PSPH), and phosphoserine aminotransferase (PSAT1) enhance production of l-serine (60), an endogenous activator of PKM2 (61). This process is tied to levels of NAD^+^ and glutamate. Two sets of proteins act to restore NAD^+^ and provide substrate for these enzymes. In the first, elevated SLC3A2 couples to elevated SLC7A8 as well as SL7A5, SLCA6, and SLCA7 to facilitate their surface expression. The SLC7A5 and SLC7A8 form neutral system l amino acid transporters. SLC7A6 and SLC7A7 form neutral or cationic amino acid transporters. Elevated surface expression of these transporters results in influx of glutamine (62), and elevated glutaminases (specifically, GLS and GLS2) use the glutamine to fill cellular glutamate pools (63). Second, increases in glutamic-oxaloacetic transaminase 1 (GOT1) may help facilitate production of cytosolic OAA from aspartate and conversion of α-ketoglutarate produced by PSAT1 back to glutamate (64). As part of the malate–aspartate shuttle, the OAA produced would be converted to malate by malate dehydrogenase 1, regenerating the NAD^+^ required to run the serine synthesis pathway and glycolysis (64, 65). The process may be especially significant for the β cells, because their lacking monocarboxylate transporters diminishes their ability to use lactate conversion to sustainably regenerate NAD^+^ and run glycolysis, as can happen in other pancreatic endocrine cells (65–67). Collectively, these alterations would increase glycolysis and serine synthesis to enhance the substrate flux and activity of PKM2, resulting in increased flux through the PEP cycle.

Other enzymes link amino acid metabolism to mitochondrial PEP production. Elevated levels of the aforementioned surface amino acid transporters allow entry of glutamine, leucine, valine, and isoleucine (62). Metabolism of leucine and isoleucine generates acetyl-CoA (68), which activates PCX (69) and inhibits PDH (70), further enhancing conversion of pyruvate to OAA. Increased levels of glutaminases (GLS and GLS2) increase glutamate (63), resulting in its deamination to α-ketoglutarate, which is converted to OAA, further augmenting PEP production. Leucine metabolism enhances this process by overcoming GTP inhibition of GDH (36, 59). Increased ornithine aminotransferase can also synthesize α-ketoglutarate from glutamate (71). Valine and isoleucine can also be metabolized to yield proprionyl-CoA, which can enter the TCA cycle at the succinyl-CoA step, ultimately increasing OAA and PEP concentrations (68). Enzymes involved in metabolism of serine and folate (SHMT2, ALDH1L2*,* MTHFD2, and FOLR1; Supplemental Figure 7) may alter TCA cycling through production of redox intermediates (72, 73). Finally, elevated mitochondrial PEPCK (PCK2) increases conversion of OAA to PEP (22, 23, 37, 74). Collectively, these enzyme changes support a model whereby the loss of *Zfp148* results in increased mitochondrial PEP production and flux through pyruvate kinase, with increased ATP, resulting in more rapid closure of K_ATP_ channels and enhanced insulin secretion.

Glutamine alone is insufficient to stimulate insulin secretion in conditions of low glucose, an effect overcome with increased activity of GDH (53–55). Although the Ca^2+^ responses in the amino acid–imaging experiments like those in Figure 2 are strongly influenced by modulation of PEP cycling (see *Pck2* KOs, refs. 23 and 37), we cannot rule out contributions of alternate effects of some of the amino acids in the mix used (GPCRs, depolarization; see refs. 51, 52, 75, and 76). Application of glutamine with the GDH activator BCH in conditions of low glucose provides mitochondrial PEP cycling substrate through OAA in the absence of confounding input from glycolysis or alternate effects of other amino acids. Consistent with our hypothesis of enhanced PEP cycling in β-*Zfp148*^KO^ islets, we found that these islets secrete dramatically more insulin than their control counterparts in response to glutamine with GDH activation (Figure 5 and Supplemental Figure 6). Although we could not identify through the study itself where in the PEP cycle the process is amplified, our findings point to a key role of mitochondrial PEP in the increased insulin secretion of β-*Zfp148*^KO^ islets.

A limitation of our study is that we were unable to directly measure key metabolites or their fluxes through these pathways in β-*Zfp148*^KO^ islets, because of the limited amount of islet tissue available from mice. We have previously shown that the administration of BCH alone in low-glucose conditions is insufficient to stimulate insulin secretion in islets of C57BL/6J mice and, therefore, requires a source of cellular glutamate (53). The difference in BCH and glutamine-stimulated insulin secretion thus provides an orthogonal approach for evaluating differential activity of this pathway in our β-*Zfp148*^KO^ islets.

### ZFP148-regulated pathways may be novel therapeutic targets in diabetes.

A challenge of existing therapies to enhance insulin secretion is that methods to trigger persistent secretion (e.g., sulfonylureas) frequently result in β cell exhaustion (77). In these cases, hypersecretion is followed by a decline in insulin production that, if left unchecked, can lead to β cell failure. Efforts to improve glucose sensitivity using glucokinase activators can similarly result in β cell dysfunction after prolonged use, likely due to the prolonged and elevated β cell workload and loss of pulsatile insulin secretion (78, 79).

Loss of β cell *Zfp148* mimics some mouse models of insulin hypersecretion (i.e., the shift in glucose sensing and elevated Ca^2+^ response) (80). However, in contrast to these models, β-*Zfp148*^KO^ mice remain highly glucose tolerant and preserve insulin secretion capacity and first-phase insulin release, even in the presence of islet stressors such as a high-sucrose or a high-fat WD (5). Other therapies that act on the PEP cycling pathway have shown promise in their ability to stimulate secretion while avoiding the excess workload present with glucokinase-activator therapies and persistently elevated Ca^2+^ levels (22, 23). It is possible that increased sensitivity to anaplerotic fuels that results in enhanced PEP cycling may enhance the resilience of β cells in the β-*Zfp148*^KO^ mice in the context of the dietary stress.

A limitation of our work is that the β-*Zfp148*^KO^ mice are a genetic model in which *Zfp148* was deleted late in islet development (i.e., concurrent with insulin expression) (81). Therefore, this may not reflect the physiological outcomes of acute inhibition with small-molecule therapies in adult animals. Additional studies in which the inducible, β cell–specific Cre recombinase models are used (e.g., Ins-Cre^ERT^; ref. 81) or acute knockdown of *Zfp148* with antisense oligonucleotides conjugated to GLP-1 (82) will help us differentiate acute from long-term suppression of β cell ZFP148.

Our Ca^2+^ imaging assays and BCH perifusions revealed differences in sensitivity that were not observed in our initial static-secretion measurements (5). Although this potentially speaks to the greater sensitivity of the imaging assay and perifusion in analyzing islet function, it raises the question of why this shift in sensitivity did not result in changes in basal glucose. Underlying mechanisms (e.g., altered insulin sensitivity or changes in glucagon secretion) are areas we will explore in future studies.

Our analysis focused on several genes with likely direct links to metabolic cycling and insulin secretion. These alterations are consistent with the observed changes in islet Ca^2+^ and insulin secretion. Our data clearly demonstrate altered Ca^2+^ cycling and improved sensitivity to glucose and amino acids in β-*Zfp148*^KO^ islets. The enhanced sensitivity to amino acids and the enzymes that are differentially expressed in the β-*Zfp148*^KO^ islets phenotypically converge on PEP cycling, even if not all of the PEP cycle enzymes (e.g., PKM1, PKM2, PKLR, PCX) are themselves altered. Thus, understanding how ZFP148 regulates its downstream targets may uncover attractive targets for therapies aimed at enhancing β cell function.

## Methods

### Chemicals.

All salts, glucose, BSA, DMSO, BCH, and amino acids were purchased from Sigma Aldrich. RPMI 1640 base medium (11 mM glucose) was purchased from ThermoFisher (catalog 11-875-093). Heat-inactivated FBS was purchased from Sigma (catalog 12306C). Antibiotic–antimycotic solutions were purchased from ThermoFisher (catalog 15240112). The pyruvate kinase activator TEPP-46 was purchased from MedChemExpress (catalog HY-18657). Stocks of TEPP-46 were prepared at 50 mM in DMSO, aliquoted into light-shielded tubes, and stored at –80°C until use (10 μM final concentration). Fura Red Ca^2+^ imaging dye was purchased from ThermoFisher (catalog F3020). Fura Red stocks were prepared at 5 mM concentrations in DMSO, aliquoted into light-shielded tubes, and stored at –20°C until day of use (2.5 μM final concentration). The lipid dye DiR was purchased from ThermoFisher (catalog D12731), prepared in DMSO at 2 mg/mL, aliquoted to light-shielded tubes, and stored at 4°C until use. All imaging solutions were prepared in HEPES-buffered imaging medium (see Table 2 for the formula). Amino acids were prepared as 100× stock in the HEPES-buffered imaging medium (formulas for final amino acid concentrations are given in Table 2), aliquoted into 1.5 mL tubes, and frozen at –20°C until day of use. Imaging and media solutions were adjusted to pH 7.4. In conditions where the TEPP-46 was added to the imaging solutions, equivalent DMSO (0.1%) was added to the solutions without TEPP-46 as control.

### Mice.

β-*Zfp148*^KO^ and control littermate mice were generated as previously described (5). Mice were maintained on either a chow diet (Purina 5008 or were fed a high-fat, high-sucrose WD (consisting of 44.6% kcal fat, 34% carbohydrate, and 17.3% protein) from Envigo Teklad (TD.08811) beginning at weaning and continued until sacrifice (aged ~24–28 weeks). Chow mice were sacrificed at 24–28 weeks of age to match the age range of the WD-fed animals, except for the mice used for the perifusion study, which were 17–19 weeks old. All comparisons for any imaging experiment and the insulin secretion perifusion were done between β-*Zfp148*^KO^ and littermate controls. We previously reported that male and female β-*Zfp148*^KO^ mice showed enhanced glucose tolerance and insulin secretion (5). Where possible, we performed all experiments on islets from both male and female mice. We observed no difference in birth rates for male or female β-*Zfp148*^KO^ mice. For each group, except those for amino acid studies on chow-fed male mice, animals from at least 3 litters were analyzed. The numbers of mice and islets from each that were imaged are indicated in Table 1. We confirmed all genotypes prior to experiments using PCR, as previously described (5). Animals were sacrificed by cervical dislocation prior to the islet isolations.

### Ex vivo islet imaging experiments.

Islets were isolated as previously described (53). Islets were incubated in RPMI medium containing 10% FBS and 1% antibiotic/antimycotic for 3 days prior to imaging experiments. We found the longer culture period optimal for signal measurement in these studies, though it differs from the 2-hour and overnight incubations used in the static and perifusion insulin-secretion assays, respectively, for which loss of Zfp148 has shown enhanced secretion (5) (Figure 5). Islets from β-*Zfp148*^KO^ or from control mice were imaged simultaneously. To differentiate animal of origin for islets in each experiment, islets from 1 mouse were loaded with near-infrared dye (1 μg/mL DiR in medium) for 10 minutes (37, 83). These islets were then washed in DiR-free medium, moved to a separate dish containing islets from the mouse’s littermate, followed by incubation of all islets with Fura Red (2.5 μM in medium) at 37°C for 45 minutes. Control and β-*Zfp148*^KO^ islets were alternately loaded with DiR, although we observed no effect of the DiR loading on islet Ca^2+^ responses (an example is shown in Supplemental Figure 8).

After loading, islets were placed in a glass-bottomed, open-air perfusion chamber (RC-41LP; Warner Instruments) with basal imaging solution (3 mM glucose in HEPES-buffered imaging medium; the formula is given in Table 2). To enhance our ability to measure intracellular islet Ca^2+^ dynamics, we used a slightly higher Ca^2+^ concentration (5 mM) than is typically added to Krebs-Ringer buffer (2.52 mM). The imaging chamber was placed on a 33°C–heated microscope stage (TC-344C temperature controller; Warner Instruments, which also controlled the temperature of the perfused solution via an in-line heater) of a Nikon Ti-Eclipse inverted microscope. Solution was perfused through the chamber at 0.25 mL/min, with constant flow controlled by a Fluigent MCFS-EZ and M-switch valve assembly (Fluigent). The scope was equipped with ×20/0.75NA SuperFluor objective (Nikon Instruments), a Sola SEII 365 LED light engine (Lumencor) set to 10% output, ET-type excitation and emission filters (Chroma Technology), and a FF444/521/608-Di01 dichroic beamsplitter (Semrock). Excitation/emission data are as follows: Fura Red (430/20 nm and 500/20 nm excitation, 630/70 nm emission; ratio defined as R500/430), NAD(P)H (365/20 nm excitation, 470/24 nm emission), and DiR (748 nm excitation, 780 nm emission). Time-lapse images were acquired by a Hamamatsu ORCA-Flash4.0 V2 Digital CMOS camera at 6-second intervals. A single region of interest was used to collect the average responses of each islet. Images were processed as described in the extended data in Dryad (46). All analysis script sets are available in the extended data in Dryad (46), which also contains citations for the base packages they use.

### Ex vivo islet perifusion.

Isolated islets were kept in RPMI-based medium (see above) for 24 hours prior to perifusion, which was performed as previously described with minor modifications (ref. 84; see also extended data in Dryad, ref. 46). Perifusion solutions were set to a flow rate of 100 μL/min and 100 μL samples were collected every minute after 55 minutes of equilibration in the flowing BCH-free medium. All solutions and islet chambers were kept at 37°C. At the end of sample collection, chambers were disconnected, inverted, and flushed with 2 mL of acid-ethanol (76.9% ethanol with 0.185 M HCl) for islet insulin extraction.

### Secreted insulin assay.

Insulin in each perifusion fraction and islet insulin content were determined using a custom assay, as previously described (85).

### Islet RNA isolation and processing.

Freshly isolated islets from WD-fed mice of both sexes and genotypes were collected, RNA from these islets was processed, and libraries were prepared as described in extended data in Dryad (46). Sequencing was done at the University of Wisconsin-Madison Biotechnology Center. Reads were aligned back to the mm10 mouse genome build using the short read aligner Bowtie (86) followed by RSEM (87). The resulting sequencing data were analyzed by EBSeq to determine significantly differently expressed genes (FDR = 0.05) (88). Gene ontology enrichment of differently expressed genes in the RNA-Seq data set was done using Enrichr (47, 48), and results of this analysis are included in extended data in Dryad (46). A heat map for the RNA-Seq analysis (Figure 2) was made using R. Additional information on EBSeq analysis can be found in extended data in Dryad (46).

### Determining candidate ZFP148 targets.

These analyses used ZNF148 ChIP-Seq [GEO accessions GSE105932 and GSE136444 from the ENCODE Project (44) in HEK and K562 cells, respectively] and human islet transcription factor and HiC data sets from Miguel-Escalada et al. (45). DE orthologs were considered putative targets if they had ZNF148 binding peaks within 1 kbp of their TSS, within their introns, or within 500 bp of HiC loops mapping to the DE ortholog’s TSS. Determining overlap of these data was done using R scripts.

### Isolated islet proteomics.

Additional details of the sample preparation, run, and analysis are included in extended data in Dryad (46). Mass spectrometry sample preparation followed the streamlined tandem mass tag (TMT) workflow (89). All the islet samples were fitted in a TMTpro16-plex experiment. Islets were needle lysed using 8 M urea in 100 mM EPPS (pH 8.5) with protease inhibitors. We used 1 μL of lysis buffer per 1.5 islets was used on the basis of information provided (extended data in Dryad, ref. 46). Lysates were reduced with 5 mM TCEP [tris(2-carboxyethyl)phosphine], alkylated with 10 mM iodoacetamide (dark), and quenched with 10 mM DTT (all for 20 minutes). SinglePot, solid-phase–enhanced sample processing, as described previously (90), was used during protein isolation and digestion. The samples were mixed at a 1:1 ratio across all channels on the basis of a pilot ratio check liquid chromatography–mass spectrometry experiment. The pooled, multiplexed samples were desalted using a 100 mg SepPak cartridge, of which 300 μg of peptide was fractionated via basic pH reversed-phase HPLC, collected in a 96-well plate, and concatenated into 24 fractions prior to desalting and liquid chromatography–tandem mass spectrometry analysis (91).

Mass spectrometric data were collected on an Orbitrap Fusion Lumos mass spectrometer coupled to a Proxeon NanoLC-1200 liquid chromatograph. The 100 μm capillary column was packed with 35 cm of Accucore 150 resin (2.6 μm, 150 Å; Thermo Fisher Scientific). The scan sequence began with an MS1 spectrum (Resolution, 60,000; 400–1600 Th; automatic gain control target, 400,000; maximum injection time, 50 ms). Data were acquired using the FAIMS Pro interface with 3 compensation voltages (–40, –60, and –80 V) with each scan cycle set as a 1 second TopSpeed method. Higher-energy collision dissociation (collision energy, 37%; automatic gain control target, 125,000; maximum injection time, 86 ms; resolution, 50,000) was used for MS2 fragmentation and quantification analysis.

Mass spectra were processed using a Comet-based software pipeline (92). Mass spectra raw files were converted to pepXML for processing. Database searching included *Mus musculus* entries from UniProt. Reversed sequences of all proteins were appended to the search database for the target-decoy FDR analysis (93). Searches were performed using a 50-ppm precursor ion tolerance and 0.02 Da for product ion tolerance to maximize sensitivity in conjunction with Comet searches. Peptide-spectrum match (PSM) filtering was performed using a linear discriminant analysis, as described previously (94). TMTpro tags on lysine residues and peptide N termini (+304.207 Da) and carbamidomethylation of cysteine residues (+57.021 Da) were set as static modifications, and oxidation of methionine residues (+15.995 Da) was set as a variable modification. PSMs were adjusted to a 1% FDR. Peptide intensities were quantified by summing reporter ion counts across all matching PSMs to give greater weight to more intense ions (95). We required a TMT reporter ion summed signal-to-noise (S/N) of greater than 100. Reporter ion intensities were adjusted to correct for isotopic impurities of the different TMTpro reagents according to manufacturer specifications. The S/N measurements of peptides assigned to each protein were summed, and these values were normalized so the sum of the signal for all proteins in each channel was equivalent, thereby accounting for equal protein loading (column normalization). Finally, each protein’s abundance was scaled to a percentage of the total, such that the summed S/N for that protein across all channels equaled 100, thereby generating relative abundance measurements.

### Statistics.

Ca^2+^ responses for glucose ramps were analyzed by Šidák post tests following 2-way ANOVA for each glucose concentration, with *P* values adjusted for multiple comparisons. Nonlinear fits (sigmoidal dose response, 4 parameter) were used to determine each group’s glucose response curve and the comparison between the fit parameters (bottom, EC_50_, Hill slope, and top) was determined by an extra sum-of-squares *F* test to assess whether the dose responses were different between control and β-*Zfp148*^KO^ mouse islets. AUC measures for the amino acid Ca^2+^ studies and the BCH perifusion were analyzed by Tukey or Šidák post tests following 1-way or 2-way ANOVA as appropriate, with *P* values adjusted for multiple comparisons (adjusted *P* < 0.05 was considered significant).

### Data availability.

Excel files with raw region of interest trace data, processed data files, and scripts are provided in extended data in Dryad (46). Gene expression data are deposited in the Gene Expression Omnibus with the identifier GSE182311. RNA-Seq data, gene ontology for DE genes, and other procedural data are provided in extended data in Dryad (46). Extended data in Dryad are available via the research repository Dryad (46). Raw image files are available on request.

### Study approval.

All protocols were approved by the University of Wisconsin-Madison IACUC (Protocol A005821-R01).

## Author contributions

CHE conceived experiments, performed experiments, performed data analysis, wrote the paper, and edited the paper. EK performed experiments and edited the manuscript. KLS, KM, MER, SPS, SLL, HRF, XL, JAP, QY, and DSS performed experiments. ZW, XL, and MAK performed data analysis. CMK and JCP edited the manuscript. RLC, RGK, SPG, MPK, MH, MJM, and ADA conceived the experiments and edited the manuscript.

## Supplementary Material

Supplemental data

## Figures and Tables

**Table 1:**
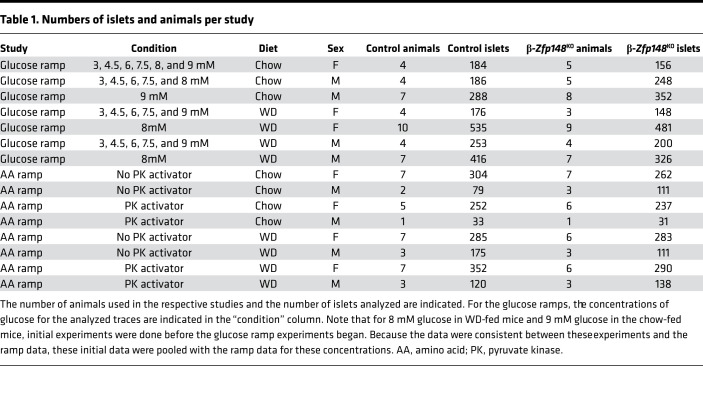
Numbers of islets and animals per study

**Table 2:**
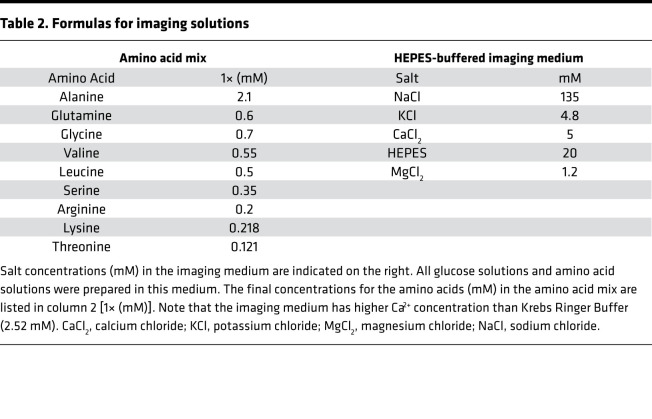
Formulas for imaging solutions

## Version 1 05/23/2022

Electronic publication

## Version 2 07/18/2022

Qing Yu's name was spelled incorrectly

## References

[1] Billings LK, Florez JC (2010). The genetics of type 2 diabetes: what have we learned from GWAS?. Ann N Y Acad Sci.

[2] Mohlke KL, Boehnke M (2015). Recent advances in understanding the genetic architecture of type 2 diabetes. Hum Mol Genet.

[3] Prasad RB, Groop L (2015). Genetics of type 2 diabetes-pitfalls and possibilities. Genes (Basel).

[4] Saul MC (2019). High-diversity mouse populations for complex traits. Trends Genet.

[5] Keller MP (2019). Gene loci associated with insulin secretion in islets from nondiabetic mice. J Clin Invest.

[6] Li X (2014). Stress hematopoiesis is regulated by the Krüppel-like transcription factor ZBP-89. Stem Cells.

[7] Sayin VI (2013). Zfp148 deficiency causes lung maturation defects and lethality in newborn mice that are rescued by deletion of p53 or antioxidant treatment. PLoS One.

[8] Takeuchi A (2003). Heterozygosity with respect to Zfp148 causes complete loss of fetal germ cells during mouse embryogenesis. Nat Genet.

[9] Passantino R (1998). Negative regulation of beta enolase gene transcription in embryonic muscle is dependent upon a zinc finger factor that binds to the G-rich box within the muscle-specific enhancer. J Biol Chem.

[10] Boopathi E (2004). Regulation of murine cytochrome c oxidase Vb gene expression during myogenesis: YY-1 and heterogeneous nuclear ribonucleoprotein D-like protein (JKTBP1) reciprocally regulate transcription activity by physical interaction with the BERF-1/ZBP-89 factor. J Biol Chem.

[11] Ohneda K (2009). Characterization of a functional ZBP-89 binding site that mediates Gata1 gene expression during hematopoietic development. J Biol Chem.

[12] Woo AJ (2011). Role of ZBP-89 in human globin gene regulation and erythroid differentiation. Blood.

[13] Sayin VI (2014). Loss of one copy of Zfp148 reduces lesional macrophage proliferation and atherosclerosis in mice by activating p53. Circ Res.

[14] Cai MY (2012). High-expression of ZBP-89 correlates with distal metastasis and poor prognosis of patients in clear cell renal cell carcinoma. Biochem Biophys Res Commun.

[15] Nilton A (2016). Targeting Zfp148 activates p53 and reduces tumor initiation in the gut. Oncotarget.

[16] Zhang CZ (2010). Transcription factor ZBP-89 in cancer growth and apoptosis. Biochim Biophys Acta.

[17] Essien BE (2016). Transcription factor ZBP-89 drives a feedforward loop of β-catenin expression in colorectal cancer. Cancer Res.

[18] Law DJ (2006). Intestinal overexpression of ZNF148 suppresses ApcMin/+ neoplasia. Mamm Genome.

[19] Chen GG (2009). ZBP-89 reduces the cell death threshold in hepatocellular carcinoma cells by increasing caspase-6 and S phase cell cycle arrest. Cancer Lett.

[20] Chen GG (2003). Mutation of p53 in recurrent hepatocellular carcinoma and its association with the expression of ZBP-89. Am J Pathol.

[21] Stark R (2009). Phosphoenolpyruvate cycling via mitochondrial phosphoenolpyruvate carboxykinase links anaplerosis and mitochondrial GTP with insulin secretion. J Biol Chem.

[22] Lewandowski SL (2020). Pyruvate kinase controls signal strength in the insulin secretory pathway. Cell Metab.

[23] Abulizi A (2020). Multi-tissue acceleration of the mitochondrial phosphoenolpyruvate cycle improves whole-body metabolic health. Cell Metab.

[24] Henquin JC (2009). Regulation of insulin secretion: a matter of phase control and amplitude modulation. Diabetologia.

[25] Tornheim K (1997). Are metabolic oscillations responsible for normal oscillatory insulin secretion?. Diabetes.

[26] Bertram R (2007). Metabolic and electrical oscillations: partners in controlling pulsatile insulin secretion. Am J Physiol Endocrinol Metab.

[27] Lang DA (1979). Cyclic oscillations of basal plasma glucose and insulin concentrations in human beings. N Engl J Med.

[28] Nunemaker CS (2006). Glucose modulates [Ca2+]i oscillations in pancreatic islets via ionic and glycolytic mechanisms. Biophys J.

[29] Jahanshahi P (2009). Evidence of diminished glucose stimulation and endoplasmic reticulum function in nonoscillatory pancreatic islets. Endocrinology.

[30] Bertram R (2018). Closing in on the mechanisms of pulsatile insulin secretion. Diabetes.

[31] Bertram R (2010). Electrical bursting, calcium oscillations, and synchronization of pancreatic islets. Adv Exp Med Biol.

[32] Merrins MJ (2010). Metabolic oscillations in pancreatic islets depend on the intracellular Ca2+ level but not Ca2+ oscillations. Biophys J.

[33] Liu YJ (1998). Origin of slow and fast oscillations of Ca2+ in mouse pancreatic islets. J Physiol.

[34] Fridlyand LE (2010). Bursting and calcium oscillations in pancreatic beta-cells: specific pacemakers for specific mechanisms. Am J Physiol Endocrinol Metab.

[35] Kennedy RT (2002). Metabolic oscillations in beta-cells. Diabetes.

[36] Smith HQ (2019). Glutamate dehydrogenase, a complex enzyme at a crucial metabolic branch point. Neurochem Res.

[38] Anastasiou D (2012). Pyruvate kinase M2 activators promote tetramer formation and suppress tumorigenesis. Nat Chem Biol.

[39] Zou ZV (2020). Genomic profiling of the transcription factor Zfp148 and its impact on the p53 pathway. Sci Rep.

[40] Law DJ (1998). The human ZBP-89 homolog, located at chromosome 3q21, represses gastrin gene expression. Mamm Genome.

[41] Merchant JL (1996). ZBP-89, a Krüppel-like zinc finger protein, inhibits epidermal growth factor induction of the gastrin promoter. Mol Cell Biol.

[42] Park H (2003). The zinc finger transcription factor ZBP-89 is a repressor of the human beta 2-integrin CD11b gene. Blood.

[43] Salmon M, Zehner ZE (2009). The transcriptional repressor ZBP-89 and the lack of Sp1/Sp3, c-Jun and Stat3 are important for the down-regulation of the vimentin gene during C2C12 myogenesis. Differentiation.

[44] ENCODE Project Consortium (2012). An integrated encyclopedia of DNA elements in the human genome. Nature.

[45] Miguel-Escalada I (2019). Human pancreatic islet three-dimensional chromatin architecture provides insights into the genetics of type 2 diabetes. Nat Genet.

[47] Chen EY (2013). Enrichr: interactive and collaborative HTML5 gene list enrichment analysis tool. BMC Bioinformatics.

[48] Kuleshov MV (2016). Enrichr: a comprehensive gene set enrichment analysis web server 2016 update. Nucleic Acids Res.

[49] Ocadiz-Ruiz R (2017). ZBP-89 function in colonic stem cells and during butyrate-induced senescence. Oncotarget.

[50] Zahra K (2020). Pyruvate kinase M2 and cancer: the role of PKM2 in promoting tumorigenesis. Front Oncol.

[51] Newsholme P, Krause M (2012). Nutritional regulation of insulin secretion: implications for diabetes. Clin Biochem Rev.

[52] Pi M (2012). GPRC6A mediates the effects of L-arginine on insulin secretion in mouse pancreatic islets. Endocrinology.

[53] Rabaglia ME (2005). Alpha-ketoisocaproate-induced hypersecretion of insulin by islets from diabetes-susceptible mice. Am J Physiol Endocrinol Metab.

[54] Vetterli L (2012). Delineation of glutamate pathways and secretory responses in pancreatic islets with β-cell-specific abrogation of the glutamate dehydrogenase. Mol Biol Cell.

[55] Tanizawa Y (2002). Unregulated elevation of glutamate dehydrogenase activity induces glutamine-stimulated insulin secretion: identification and characterization of a GLUD1 gene mutation and insulin secretion studies with MIN6 cells overexpressing the mutant glutamate dehydrogenase. Diabetes.

[56] Udelhoven M (2010). Neuronal insulin receptor substrate 2 (IRS2) expression is regulated by ZBP89 and SP1 binding to the IRS2 promoter. J Endocrinol.

[57] Mazuy C (2013). Palmitate increases Nur77 expression by modulating ZBP89 and Sp1 binding to the Nur77 proximal promoter in pancreatic β-cells. FEBS Lett.

[58] Schaum N (2018). Single-cell transcriptomics of 20 mouse organs creates a Tabula Muris. Nature.

[59] Li C (2006). Effects of a GTP-insensitive mutation of glutamate dehydrogenase on insulin secretion in transgenic mice. J Biol Chem.

[60] Holm LJ, Buschard K (2019). L-serine: a neglected amino acid with a potential therapeutic role in diabetes. APMIS.

[61] Chaneton B (2012). Serine is a natural ligand and allosteric activator of pyruvate kinase M2. Nature.

[62] Bhutia YD, Ganapathy V (2016). Glutamine transporters in mammalian cells and their functions in physiology and cancer. Biochim Biophys Acta.

[63] Katt WP (2017). A tale of two glutaminases: homologous enzymes with distinct roles in tumorigenesis. Future Med Chem.

[64] Kremer DM (2021). GOT1 inhibition promotes pancreatic cancer cell death by ferroptosis. Nat Commun.

[65] Brun T, Maechler P (2016). Beta-cell mitochondrial carriers and the diabetogenic stress response. Biochim Biophys Acta.

[66] Pullen TJ, Rutter GA (2013). When less is more: the forbidden fruits of gene repression in the adult β-cell. Diabetes Obes Metab.

[67] Pullen TJ (2012). Overexpression of monocarboxylate transporter-1 (SLC16A1) in mouse pancreatic β-cells leads to relative hyperinsulinism during exercise. Diabetes.

[68] Holeček M (2018). Branched-chain amino acids in health and disease: metabolism, alterations in blood plasma, and as supplements. Nutr Metab (Lond).

[69] Jitrapakdee S (2008). Structure, mechanism and regulation of pyruvate carboxylase. Biochem J.

[70] Bremer J (1969). Pyruvate dehydrogenase, substrate specificity and product inhibition. Eur J Biochem.

[71] Ginguay A (2017). Ornithine aminotransferase, an important glutamate-metabolizing enzyme at the crossroads of multiple metabolic pathways. Biology (Basel).

[72] Yang M, Vousden KH (2016). Serine and one-carbon metabolism in cancer. Nat Rev Cancer.

[73] Yang L (2020). Serine catabolism feeds NADH when respiration is impaired. Cell Metab.

[74] Yu S (2021). Phosphoenolpyruvate carboxykinase in cell metabolism: roles and mechanisms beyond gluconeogenesis. Mol Metab.

[75] Keane K, Newsholme P (2014). Metabolic regulation of insulin secretion. Vitam Horm.

[76] Henquin J-C (2021). Paracrine and autocrine control of insulin secretion in human islets: evidence and pending questions. Am J Physiol Endocrinol Metab.

[77] Remedi MS, Nichols CG (2008). Chronic antidiabetic sulfonylureas in vivo: reversible effects on mouse pancreatic beta-cells. PLoS Med.

[78] Whitticar NB, Nunemaker CS (2020). Reducing glucokinase activity to enhance insulin secretion: a counterintuitive theory to preserve cellular function and glucose homeostasis. Front Endocrinol (Lausanne).

[79] Nakamura A, Terauchi Y (2015). Present status of clinical deployment of glucokinase activators. J Diabetes Investig.

[80] Corbin KL (2016). Islet hypersensitivity to glucose is associated with disrupted oscillations and increased impact of proinflammatory cytokines in islets from diabetes-prone male mice. Endocrinology.

[81] Thorens B (2015). Ins1(Cre) knock-in mice for beta cell-specific gene recombination. Diabetologia.

[82] Ämmälä C (2018). Targeted delivery of antisense oligonucleotides to pancreatic β-cells. Sci Adv.

[83] Capozzi ME (2019). β Cell tone is defined by proglucagon peptides through cAMP signaling. JCI Insight.

[84] Bhatnagar S (2011). Positional cloning of a type 2 diabetes quantitative trait locus; tomosyn-2, a negative regulator of insulin secretion. PLoS Genet.

[85] Mitok KA (2018). Islet proteomics reveals genetic variation in dopamine production resulting in altered insulin secretion. J Biol Chem.

[86] Langmead B (2009). Ultrafast and memory-efficient alignment of short DNA sequences to the human genome. Genome Biol.

[87] Li B, Dewey CN (2011). RSEM: accurate transcript quantification from RNA-seq data with or without a reference genome. BMC Bioinformatics.

[88] Leng N (2013). EBSeq: an empirical Bayes hierarchical model for inference in RNA-seq experiments. Bioinformatics.

[89] Navarrete-Perea J (2018). Streamlined tandem mass tag (SL-TMT) protocol: an efficient strategy for quantitative (Phospho)proteome profiling using tandem mass tag-synchronous precursor selection-MS3. J Proteome Res.

[90] Hughes CS (2014). Ultrasensitive proteome analysis using paramagnetic bead technology. Mol Syst Biol.

[91] Paulo JA (2016). Quantitative mass spectrometry-based multiplexing compares the abundance of 5000 S. cerevisiae proteins across 10 carbon sources. J Proteomics.

[92] Eng JK (2015). A deeper look into Comet--implementation and features. J Am Soc Mass Spectrom.

[93] Elias JE, Gygi SP (2010). Target-decoy search strategy for mass spectrometry-based proteomics. Methods Mol Biol.

[94] Huttlin EL (2010). A tissue-specific atlas of mouse protein phosphorylation and expression. Cell.

[95] McAlister GC (2012). Increasing the multiplexing capacity of TMTs using reporter ion isotopologues with isobaric masses. Anal Chem.

