# Supplementary material for: β Cell–specific deletion of Zfp148 improves nutrient-stimulated β cell Ca2+ responses
Source: JCI Insight. 2022 May 23;7(10):e154198. doi: 10.1172/jci.insight.154198 (PMC9220824; doi:10.1172/jci.insight.154198)
Supplement: Supplemental data [file jciinsight-7-154198-s008.pdf]

SUPPLEMENTAL INFORMATION RELATED TO:

"β-cell-specific deletion of *Zfp148* improves nutrient-stimulated β-cell Ca^{2+} responses"

Short title: "β-cell *Zfp148* inhibits nutrient-driven islet Ca^{2+} responses"

Christopher H. Emfinger[1], Eleonora de Klerk[2], Kathryn L. Schueler[1], Mary E. Rabaglia[1], Donnie S. Stapleton[1], Shane P. Simonett[1], Kelly A. Mitok[1], Ziyue Wang[3‡,4], Xinyue Liu[5], Joao A. Paulo[5], Qinq Yu[5], Rebecca L. Cardone[6], Hannah R. Foster[7], Sophie L. Lewandowski[7], José C. Perales[8], Christina M. Kendziorski[3], Steven P. Gygi[5], Richard G. Kibbey[6,9], Mark P. Keller[1], Matthias Hebrok[2], Matthew J. Merrins*[7,10] and Alan D. Attie*[1]

[1]Department of Biochemistry, University of Wisconsin-Madison, Madison, WI, 53706, USA

[2]UCSF Diabetes Center, University of California San Francisco, San Francisco, CA, 94143, USA.

[3]Department of Biostatistics & Medical Informatics, University of Wisconsin-Madison, Madison, WI, 53706, USA

[4]Biostatistics & Computational Biology Branch, National Institute of Environmental Health Sciences, Durham, NC, 27709, USA

[5]Department of Cell Biology, Harvard Medical School, Boston, MA, 02115 USA

[9]Department of Internal Medicine (Endocrinology), Yale University, New Haven, CT, USA.

[7]Department of Medicine, Division of Endocrinology, University of Wisconsin-Madison, Madison, WI, 53705, USA

[8]Department of Physiological Sciences, School of Medicine, University of Barcelona, Feixa Llarga s/n, 08907 L'Hospitalet del Llobregat, Spain

[6]Department of Cellular and Molecular Physiology, Yale University, New Haven, CT, 06520 USA

[10]William S. Middleton Memorial Veterans Hospital, Madison, WI 53705

‡ indicates previous affiliation

*Corresponding authors

For inquiries, please address correspondence to:

Alan D. Attie

543A HF DeLuca Biochemistry Laboratories, 433 Babcock Drive, Madison, WI 53706

(608) 262-1372

attie@biochem.wisc.edu

Matthew J. Merrins

C4134 William S. Middleton Memorial Veterans Hospital, 2500 Overlook Terrace, Madison, WI 53705

(716) 397-7557

merrins@wisc.edu

Supplemental file 1: ***Ontology enrichment for RNA-seq and proteomics of β-*****Zfp148*****^{KO} and control mouse islets.*** This folder contains information regarding the RNA sequencing quality control, processing, proteomic raw data, processed proteomic data, and GO enrichments. Significantly differently expressed (DE) genes or altered proteins were grouped into lists corresponding to those increased or decreased in the β-*Zfp148*^{KO} islets relative to islets from littermate control mice. These lists were entered into Enrichr (32, 33) and results incorporated here. Two additional lists, one for those genes with either a decrease or increase greater than 2.5 fold and one for genes that were designated as likely targets, were also submitted to Enrichr and the results incorporated into the file. The file also contains the processed EBSeq data as well as additional information on the cDNA preparation, on the FastQ files found on GEO, and on the EBSeq processing steps. An absolute fold change > 1.25 and p-value (adjusted for multiple comparison) of < 0.05 were used as the cutoff values for increased and decreased proteins. An included MS Excel file (.xlsx) contains an index of the archive.

Supplemental file 2: ***Scripts and raw traces used in curve analysis and figure generation***. This file contains data and the relevant scripts for the analysis of the glucose and amino acid curves. These files are organized into subfolders with a master index file (MS Excel (.xlsx)) which is included to guide those downloading the files to the relevant scripts of interest. This file also includes the raw data for the insulin ELISA for the BCH perifusion experiment (Figure 5).

Supplemental file 3: ***Extended Materials and Methods and procedures for proteomic processing and groups***. This file contains the Extended Materials and Methods as well as more detailed procedures used for the proteomics analysis, the mouse ID data for those experiments, and the summary proteomic data. These are in two MS Word files and a MS Excel workbook (.xlsx) file.

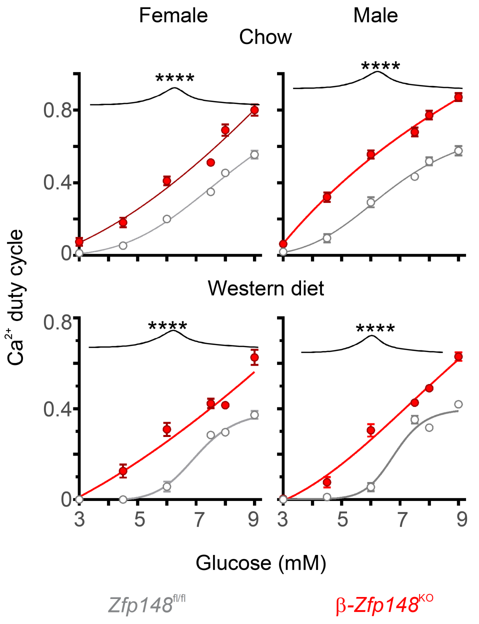

Supplementary figure 1: ***By-islet dose-response curves indicate duty cycle increase and glucose sensitivity shift in β-*****Zfp148*****^{KO} islets***. Average duty cycle dose-response for all individual islets (individual points seen in Figure 1 D) vs. glucose in chow-fed (upper) and western-diet (WD)-fed (lower) β-*Zfp148*^{KO} and littermate control mouse islets. Both females (left) and males (right) were analyzed. Individual points were compared using Sidak's multiple comparisons test following ANOVA. The bar and p value above the overall curves indicates significance of comparing the curve parameters (EC50, hill slope, and top) for the dose-response fit curves for each genotype, compared using Extra Sum-of-squares F test. For corrected p-value comparisons, **** indicates adjusted p<0.0001. Individual glucose points are graphed as mean ± SEM. Related to figure 1.

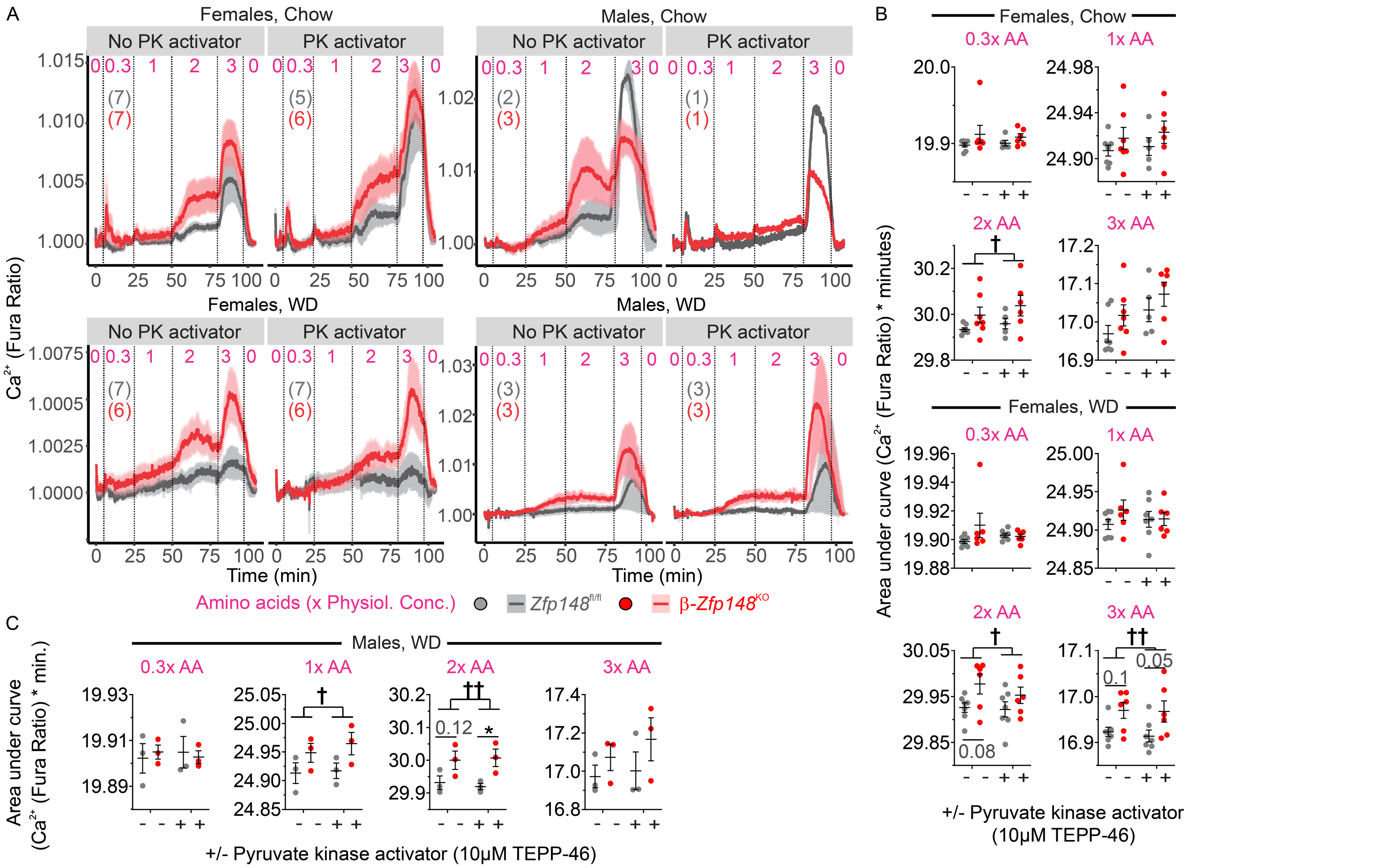

Supplementary figure 2: *By-animal grand means indicate increased amino acid sensitivity in β-*Zfp148*^{KO} mouse islets.* (**A**) Grand mean curves (an average of each animal's islets' average response) for calcium responses to a low-glucose, amino-acid ramp in in chow-fed (upper) and western-diet (WD)-fed (lower) β-*Zfp148*^{KO} and littermate control mouse islets. Females and males are on the left and right panel sets, respectively. Presence of pyruvate kinase (PK) activator (10µM TEPP-46) in the solutions is indicated in boxes above the traces. All solutions contained 3mM glucose. The concentration of mixed amino acids (AA) (relative to physiological concentration for each amino acid in the mix, where 1 is physiological concentration) is indicated above each transition in the traces. Dotted lines indicate transitions between solutions. Numbers in parentheses indicate the number of β-*Zfp148*^{KO} and littermate animals averaged for the traces. Each point is graphed with mean (solid line) ± SEM (transparent bars), except for the males with the PK activator, for which only one animal has been analyzed/group. (**B**) Area-under-the-curve (AUC) analysis for amino acid ramp traces from chow-fed (upper panels) and WD-fed (lower panels) female β-*Zfp148*^{KO} and littermate mice in the given solutions, with the concentration of AA indicated above the graphs. Points indicate by-animal grand means for islet traces and lines indicate mean ± SEM. Presence (+/-) of PK activator is indicated below the graphs. Groups were compared by Tukey's post-tests following 1-way ANOVA. (**C**) Islet average AUC analysis for WD-fed male mice. Concentrations of the AA are indicated above the graphs. For corrected p-value comparisons, * indicates $p<0.05$ for the post-test comparisons. † and †† indicate $p<0.05$ and $p<0.01$, respectively, for the gene effect as determined by 1-way ANOVA. Related to figure 2.

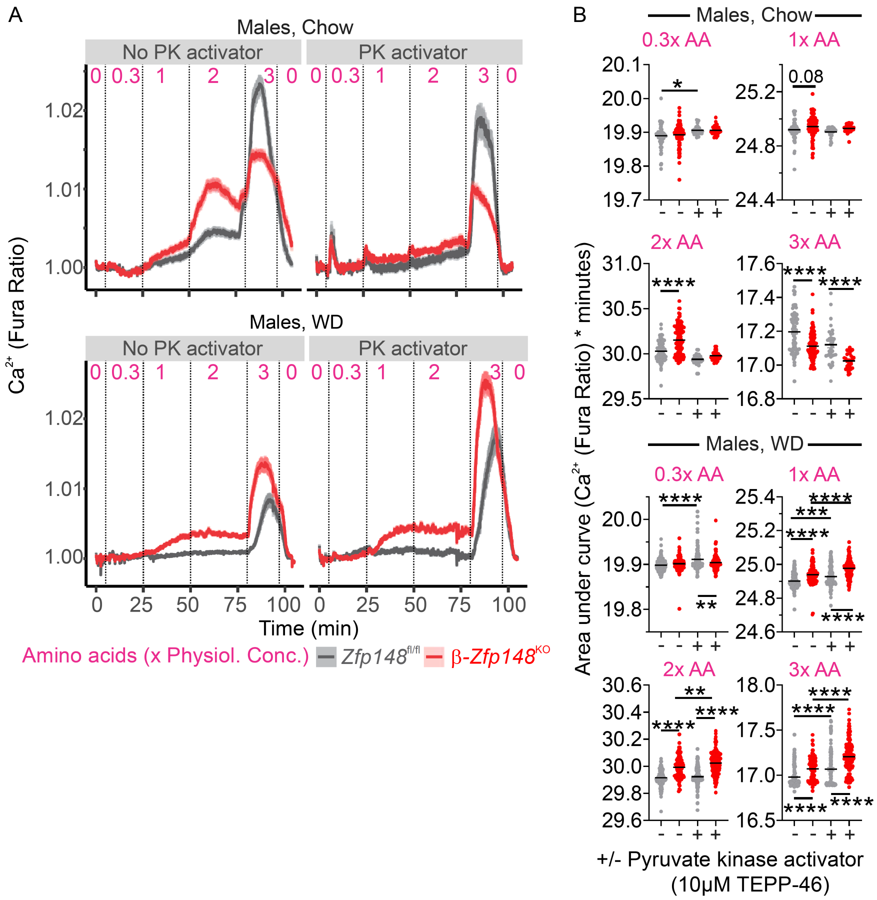

Supplementary figure 3: ***Altered amino acid sensitivity in male β-Zfp148^{KO} mouse islets.*** (**A**) Average traces for calcium responses (detrended Fura Red ratio) to a low-glucose, amino-acid ramp in in chow-fed (upper) and western-diet (WD)-fed (lower) β-*Zfp148*^{KO} and littermate control male mouse islets. Presence of pyruvate kinase (PK) activator (10µM TEPP-46) in the solutions is indicated in boxes above the traces. All solutions contained 3mM glucose. The concentration of mixed amino acids (AA) (relative to physiological concentration for each amino acid in the mix, where 1 is physiological concentration) is indicated above each transition in the traces. Transitions between solutions are indicated by vertical dotted lines at the respective times. (**B**) Area-under-the-curve (AUC) analysis for amino acid ramp traces from chow-fed (upper panels) and WD-fed (lower panels) male β-*Zfp148*^{KO} and littermate mice in the given solutions, with the concentration of AA indicated above the graphs. Points indicate individual islet trace AUCs and lines indicate means. Presence (+/-) of PK activator is indicated below the graphs. Groups were compared by Tukey's post-tests following 1-way ANOVA. (**C**) Islet average AUC analysis for WD-fed male mice. Concentrations of the concentration of AA are indicated above the graphs. For corrected p-value comparisons, *, **, ***, and **** indicate $p<0.05$, $p<0.01$, $p<0.001$, $p<0.0001$, respectively. Related to figure 2.

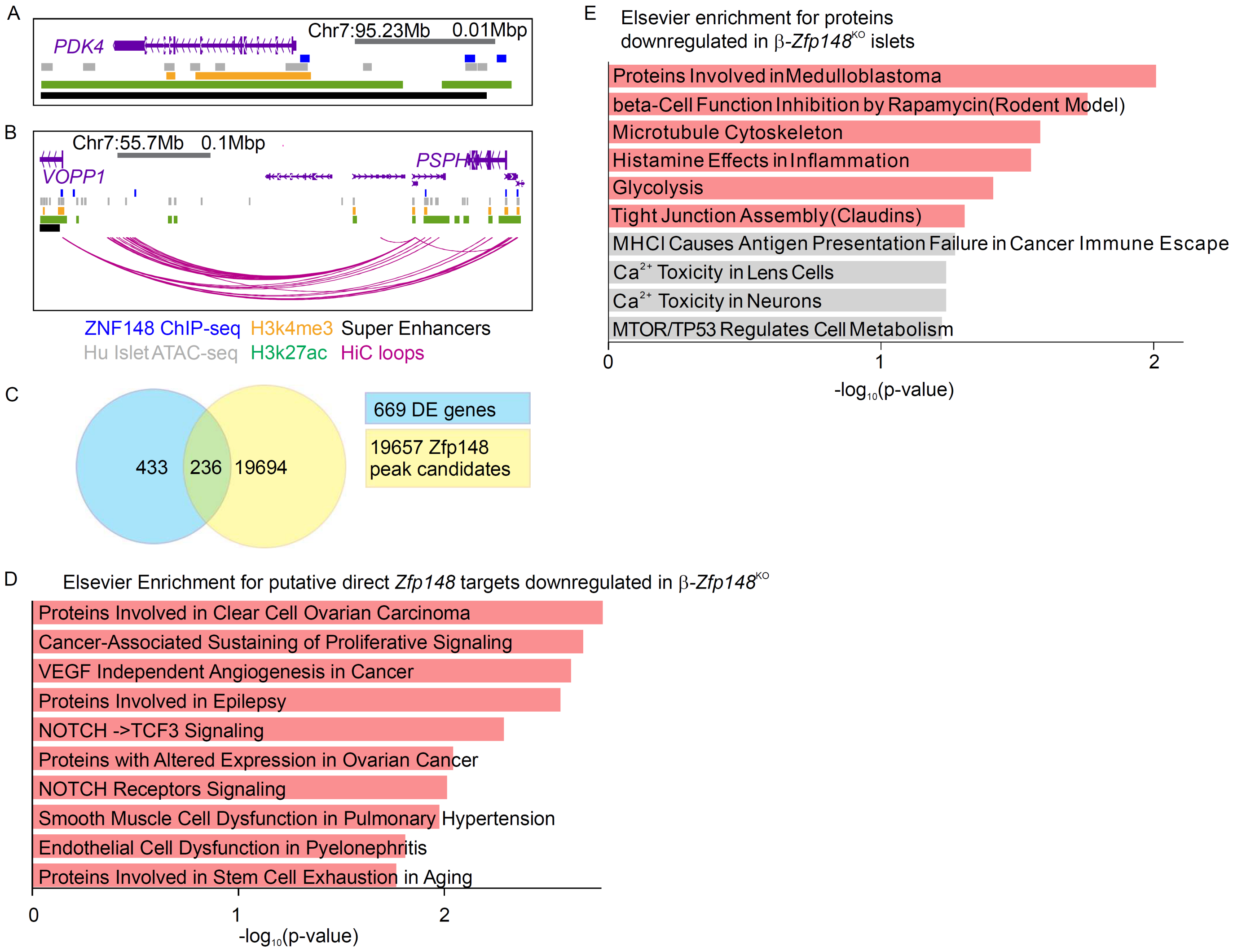

Supplementary figure 4: ***Identification of putative direct targets of Zfp148 and enrichment.*** Alignment of DE gene orthologues *PDK4* (**A**) as well as *VOPP1* and *PSPH* (**B**) with human islet chromatin data. **ZNF148 ChIP-seq peaks** from K562 and HEK cells (from (34)) align near promoters of the three genes. These are also associated with **increased islet histone methylation**, **acetylation**, and **human islet ATAC-seq peaks** and may also link the TSS to "**super enhancer**" regions either by direct alignment or, in the case of genes such as *PSPH*, aligning with chromatin loops (**HiC**) connecting the gene's promoter to the enhancer region. Human islet chromatin annotation data are from (35). (**C**) Venn diagram overlay of DE gene orthologues and ZNF148 ChIP-seq peaks identified in other cell types (from (34, 62)). (**D**) Elsevier database pathway enrichment (from Enrichr (32, 33)) for downregulated putative direct DE targets of Zfp148 based on analysis in (**A**), (**B**), and (**C**). (**E**) Elsevier database pathway enrichment for proteins significantly decreased in the β-*Zfp148*^{KO} islets. For pathway analyses, terms in **bold** indicate enrichment beyond the false discovery cutoff (adjusted p-value < 0.05). Corresponding genes and proteins for the indicated terms are listed in supplemental file 1. Related to figure 3.

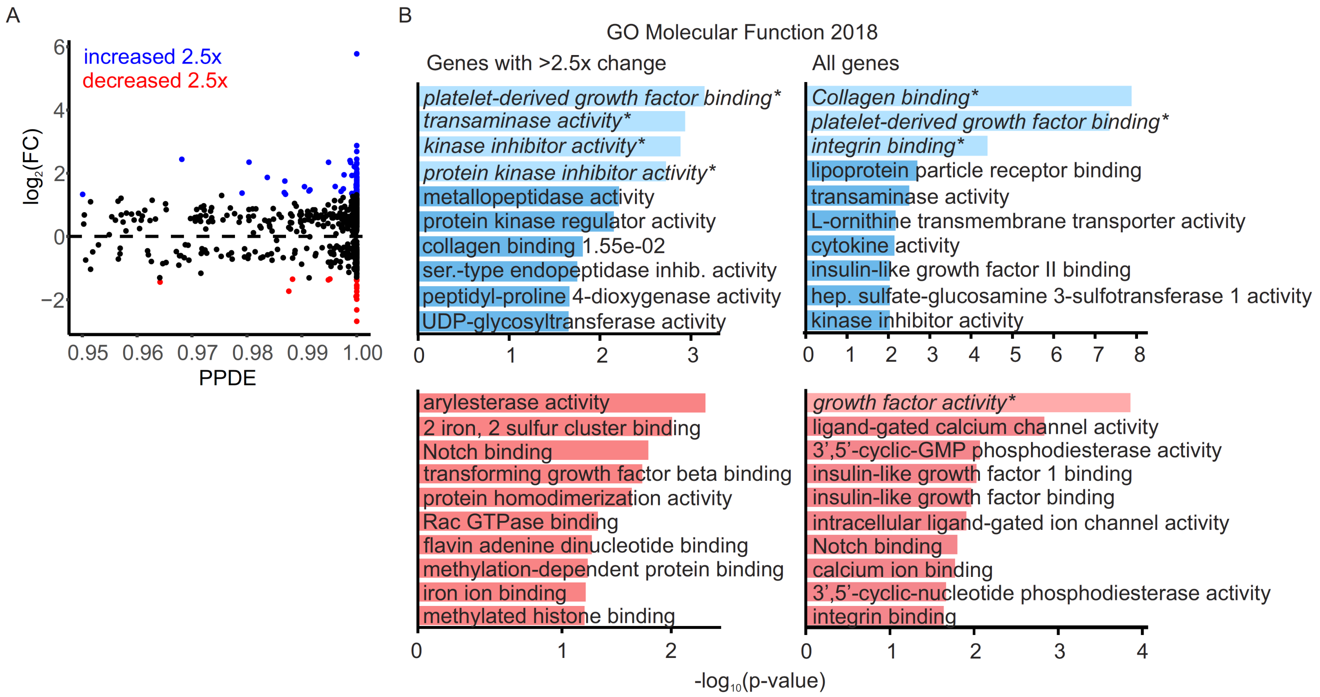

Supplemental figure 5: ***Gene ontology enrichment for all upregulated and downregulated DE genes*** (**A**) Posterior probability of differential expression (PPDE, from EBSeq) vs. $\log_2$(fold change, FC) for DE genes illustrating highly altered (FC $\geq$ 2.5 or $\leq$ -2.5) DE genes. (**B**) Molecular function gene ontology enrichment for the highly altered DE (left) or all DE (right) increased (upper) and decreased (lower) genes. Terms in *italics* indicate enrichment beyond the false discovery cutoff (adjusted p-value < 0.05). Enrichments determined using the Enrichr tool (32, 33). Terms and corresponding genes are listed in supplemental file 1. Related to figure 3.

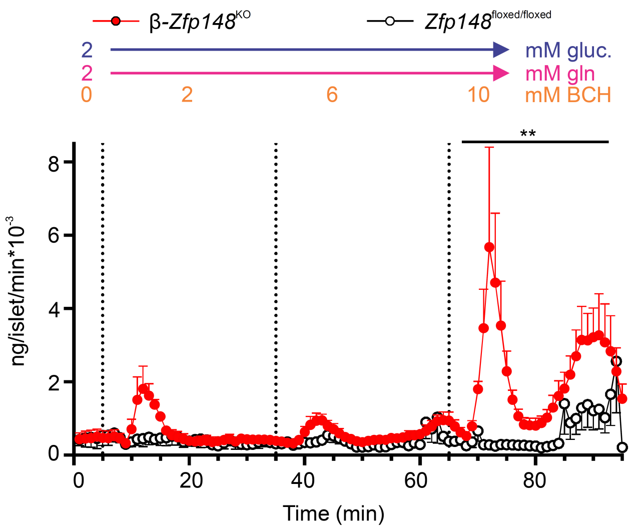

Supplementary figure 6: *β-Zfp148^{KO} mouse islets show increased raw insulin secretion in response to BCH and glutamine.* Raw insulin secretion of chow-fed male β-*Zfp148*^{KO} islets (red circles and traces) and control (open circles, black traces) during a low-glucose ramp of glutamate dehydrogenase activator BCH in perifusion. Dotted vertical lines indicate transitions between the different solutions. All solutions contained 2mM glucose and 2mM L-glutamine. Concentration (in mM) of BCH is indicated above each respsective segment of the perifusion traces. N = 4/genotype. (**) $p<0.01$, Sidak's post-test following 2-way ANOVA of area under the curve (AUC) of the segment, β-*Zfp148*^{KO} vs. control islets. Trace points display mean ± SEM. Related to figure 5.

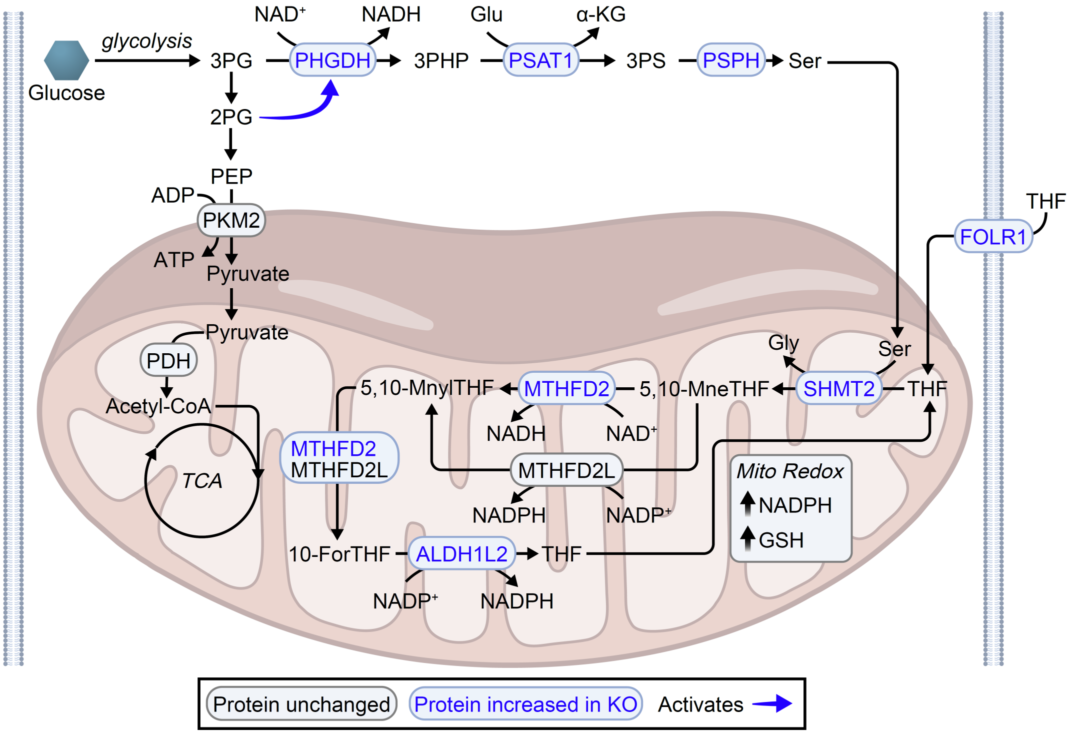

Supplementary figure 7: *β-Zfp148^{KO} mouse islets have increased mitochondrial 1-C proteins.* In this model, elevated levels of serine methyltransferase 2 (SHMT2) allows entry of L-serine into the 1-C pathway, wherein elevated methylenetetrahydrofolate dehydrogenase (NADP+ Dependent) 2 (MTHFD2), methylenetetrahydrofolate Dehydrogenase (NADP+ Dependent) 2-like (MTHFD2L), and elevated aldehyde dehydrogenase 1 family, member L2 (ALDH1L2) mediate recycling of carbon to tetrahydrofolate (THF), generating redox intermediates NADH and NADPH which can drive ATP production and glutathione cycling. Related to figure 4.

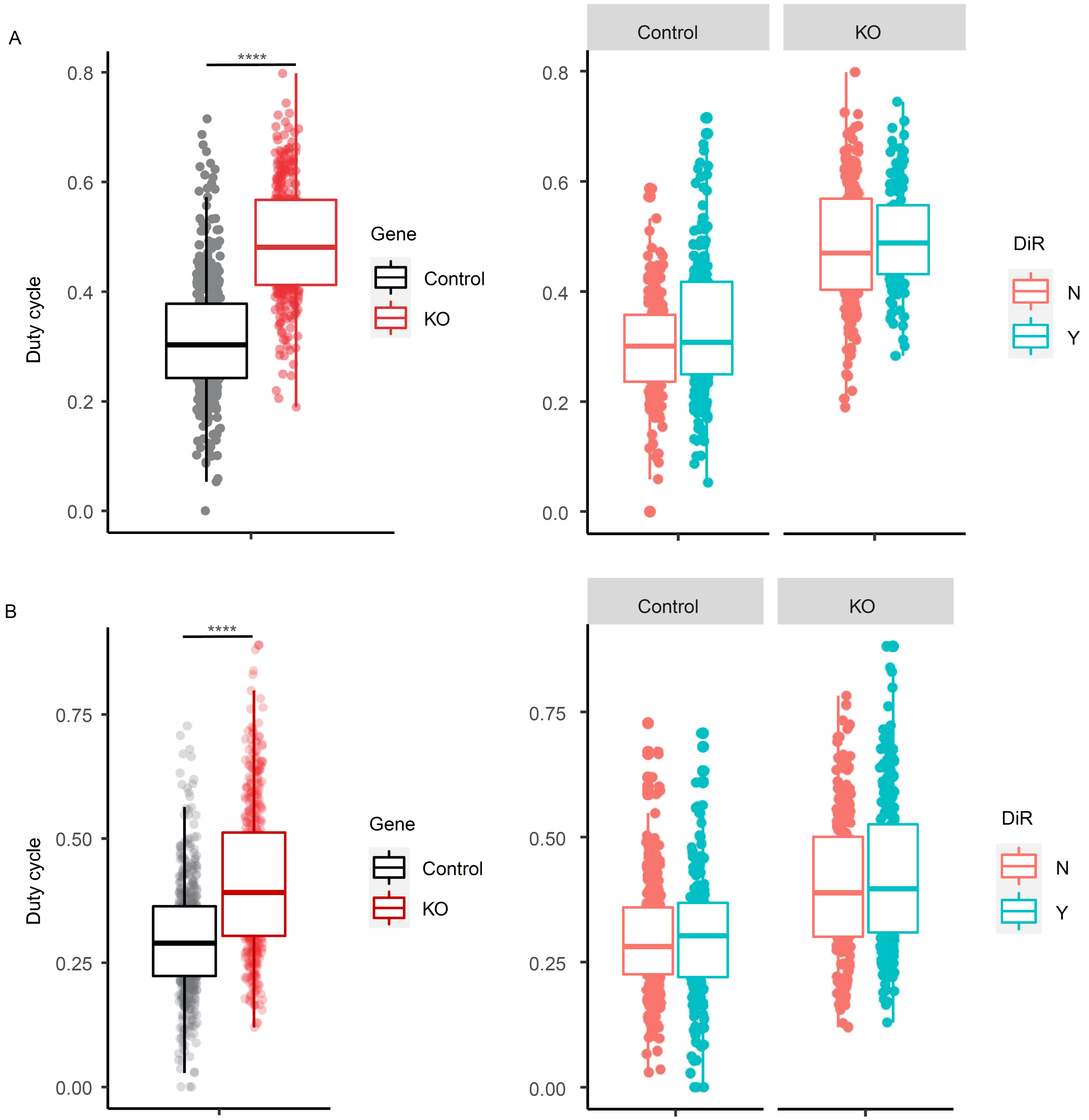

Supplemental figure 8: ***No effect of DiR on Ca^{2+} responses.*** Islet duty cycle from male (A) and female (B) WD-fed mice show duty cycle differences at 8mM glucose (left) but no differences within groups when broken down by which islets/group are DiR loaded (right). Related to figure 1.
